# Current insights in the molecular genetic pathogenesis of amyotrophic lateral sclerosis

**DOI:** 10.3389/fnins.2023.1189470

**Published:** 2023-08-10

**Authors:** Wan Zhou, Renshi Xu

**Affiliations:** ^1^Medical College of Nanchang University, Nanchang, China; ^2^Department of Neurology, Jiangxi Provincial People’s Hospital, The First Affiliated Hospital of Nanchang Medical College, The Clinical College of Nanchang Medical College, Nanchang, China

**Keywords:** amyotrophic lateral sclerosis, pathogenesis, genetics, molecule, proteomics

## Abstract

Amyotrophic lateral sclerosis (ALS) is a progressive and fatal neurodegenerative disease that leads to the massive loss of motor neurons in cerebrum, brain stem and spinal cord. It affects not only motor neurons but also other neurons and glial cells, resulting in the progressive muscle atrophy, the severe disability and the eventual death due to the respiratory failure. The pathogenesis of ALS is not fully understood. Currently, several factors are considered to be involved in the pathogenesis of ALS, such as genetic factors, imbalances in protein homeostasis, RNA metabolism disorders, mitochondrial dysfunctions, glutamate-mediated excitatory toxicities and intra-neuronal material transport disorders in neurons. The study of genetic mutations related to ALS pathogenesis will link the molecular and cellular mechanisms of the disease, thus enhancing the understanding of its occurrence and progression, thereby providing new insights for the pathogenesis of ALS. This review summarizes the current insights in the molecular genetic pathogenesis of ALS.

## 1. Introduction

Amyotrophic lateral sclerosis (ALS) is a rare chronic progressive motor neuron disease that impairs voluntary muscle control and movement. The main pathological features of ALS is the damage of motor neurons from motor cortex to neuromuscular system, including the defects of cerebrum, brain stem, spinal cord and neuromuscular junction, leading to the skeletal muscle decomposition and generalization, progressive atrophy (Fiscon et al., 2021). ALS is one of the most common neurodegenerative diseases with an incidence rate of 1 to 5 per 100,000 populations. Clinically, ALS is manifested by muscle weakness and atrophy, spasm, and contracture (Herrando-Grabulosa et al., 2021). The ALS progresses rapidly and the survival time after the onset is usually 3–5 years. The main cause of death is the respiratory failure due to the respiratory muscle paralysis and atrophy. ALS can be generally classified into both familial and sporadic ALS, the familial ALS accounts for about 5%–10% of ALS and involves the genetic mutations in genes such as superoxide dismutase 1 (*SOD1*), chromosome 9 open reading frame 72 (*C9orf72*), fused in sarcoma (*FUS*), TAR DNA-binding protein (*TDP-43/TARDBP*), and others. The sporadic ALS cases account for about 90%–95% of the disease, and the pathogenesis of ALS is not still completely clear (Je et al., 2021), which may be caused by genetics and related factors together (Fernández-Ruiz et al., 2021).

**Figure 1:**
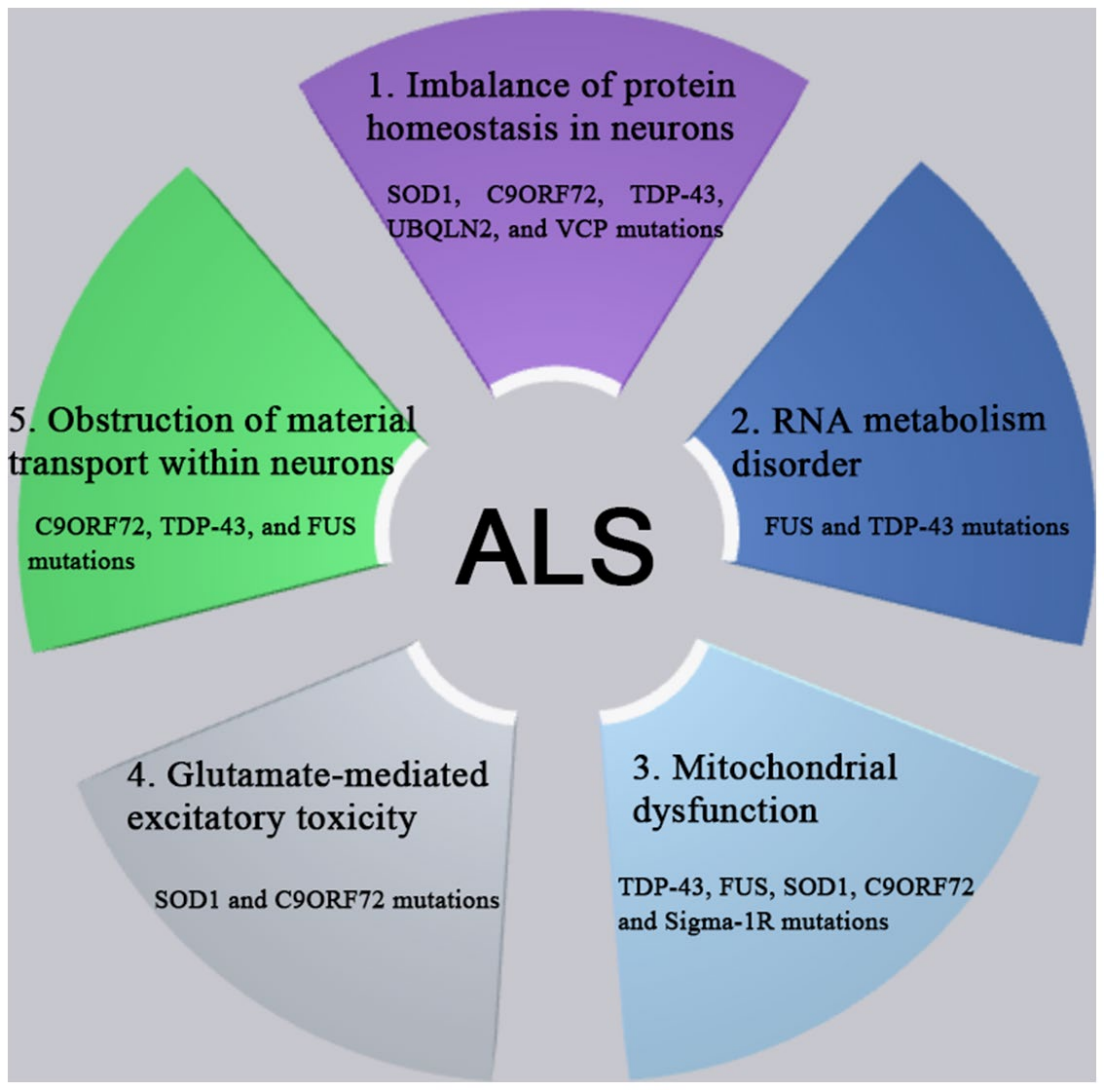
The pathogenesis of ALS involves complex processes with multiple genes and multiple pathways dysfunction, such as the imbalance of protein homeostasis in neurons, the disorder of RNA metabolism, the mitochondrial dysfunction, the glutamate-mediated excitatory toxicity and the disturbance of intra-neuronal material transport. So far, more than 50 genes have been found to be associated with ALS. Among them, *C9orf72*, *SOD1*, *TDP-43*, and *FUS* genes were most closely related to the pathogenesis of ALS. The mutations of various related genes play the different roles in the different pathways in the pathogenesis of ALS, which are interrelated and act together to lead to the occurrence and development pathogenesis of ALS.

The pathogenesis of ALS involves in the complex processes involving the multiple genes and the multiple pathways. A variety of genes and pathways (Figure 1) are involved in the pathogenesis of ALS, such as the imbalance of protein homeostasis in neurons, the disorder of ribonucleic acid (RNA) metabolism, the mitochondrial dysfunction, the glutamate-mediated excitatory toxicity and the disturbance of intra-neuronal material transport. So far, more than 50 genes have been found to be associated with ALS (Li et al., 2023). Among them, *C9orf72*, *SOD1*, *TDP-43* and *FUS* genes were most closely related to the pathogenesis of ALS (Kim et al., 2020). The mutations of various related genes play the different roles in the different pathways in the pathogenesis of ALS, which are interrelated and act together to lead to the occurrence and development pathogenesis of ALS.

Mutations in genes such as *SOD1*, *C9orf72*, *TDP-43, ubiquilin-2*, and valosin-containing protein (*VCP*) may lead to protein homeostasis imbalance through interference with the ubiquitin-proteasome system (UPS) and autophagy pathway, resulting in abnormal accumulation of toxic proteins and eventually leading to cell death (Beckers et al., 2021; Chua et al., 2022). FUS and TDP-43 are RNA-binding proteins (RBPs) associated with RNA metabolism (Hallegger et al., 2021). Mutations in these proteins cause abnormal localization and aggregation of FUS and TDP-43 in cells, affecting RNA metabolism and cellular function, ultimately leading to neurodegeneration (Ling et al., 2013). Mitochondrial dysfunction plays a crucial role in the pathogenesis of ALS as it affects multiple cellular pathways, including energy production, Ca^2+^ regulation, apoptosis, and protein quality control (Boyman et al., 2020). Mutations in ALS-associated genes such as TDP-43, FUS, SOD1, C9orf72, and Sigma-1 receptor (Sigma-1R) can alter mitochondrial integrity and function (Smith et al., 2019; Shoshan-Barmatz et al., 2020; Dafinca et al., 2021; Wood et al., 2021). Excitotoxicity is caused by excessive activation of glutamate receptors, inability to clear glutamate neurotransmitter, or increased postsynaptic sensitivity to glutamate. Glutamate transporters in astrocytes, such as excitatory amino acid transporters 1 (EAAT1)/glutamate–aspartate transporters (GLAST) and excitatory amino acid transporters 2 (EAAT2)/glial glutamate transporter-1 (GLT-1), maintain proper extracellular glutamate levels, preventing excitotoxicity. Aberrant post-translational modification pathways of EAAT1/GLAST and EAAT2/GLT-1 are associated with the pathogenesis of ALS (Diaper et al., 2013; Pajarillo et al., 2019; Hirschberg et al., 2021). Axonal transport pathways in motor neurons maintain their normal structure and function, and mutations in C9orf72, TDP-43, and FUS are associated with neuronal transport impairments (Burk and Pasterkamp, 2019; Pang and Hu, 2021).

At present, the study field of ALS continues to rapidly develop with the discovery of multiple disease related genes each year. The rare genes associated with the pathogenesis of ALS include glycosyltransferase 8 domain (*GLT8D*), carbonic anhydrase 1 (*CA1*) and serine palmitoyltransferase long chain base subunit 1 (*SPTLC1*) genes, and a series of new risk and modifying factors. A study (Cooper-Knock et al., 2019) has found a *p.R92C* mutation in the exon 4 of the gene encoding the glycosyltransferase *GLT8D1*, which is co-segregated with the disease in a family with the autosomal dominant ALS. In addition, it was discovered that the familial ALS patients exhibit a significant enrichment of additional rare deleterious variants in the exon 4 of *GLT8D1*, which clusters near the substrate-binding domain of the exon and reduces the enzyme activity of *GLT8D1* (Cao et al., 2021; Candelise et al., 2022). More and more evidence suggests that hippocampus is involved in the pathogenesis of ALS (Machts et al., 2018), the characteristic alteration in ALS is the overall volume loss and the local atrophy of the CA1 area of hippocampus, representing the neuronal correlates of the cognitive and behavioral deficits frequently encountered in this ALS disease. Recently, it has been discovered that the mutations in the *SPTLC1* subunit of serine palmitoyltransferase (SPT) catalyzes the first step of *de novo* synthesis of sphingolipids and leads to the childhood-onset ALS (Johnson et al., 2021; Mohassel et al., 2021). The variants in *SPTLC1* shifts the usage of SPT amino acid from serine to alanine, leading to the elevated levels of deoxysphingolipids and presenting as an alternating phenotype of inherited sensory and autonomic neuropathy, and being characterized by the progressive sensory loss with the varying degrees of autonomic involvement (Mohassel et al., 2021). This article provides the current insights of molecular genetic mechanisms underlying the pathogenesis of ALS based on the currently recognized relationship between genetic genes and molecular pathways.

## 2. Imbalance of protein homeostasis in neurons

**Figure 2:**
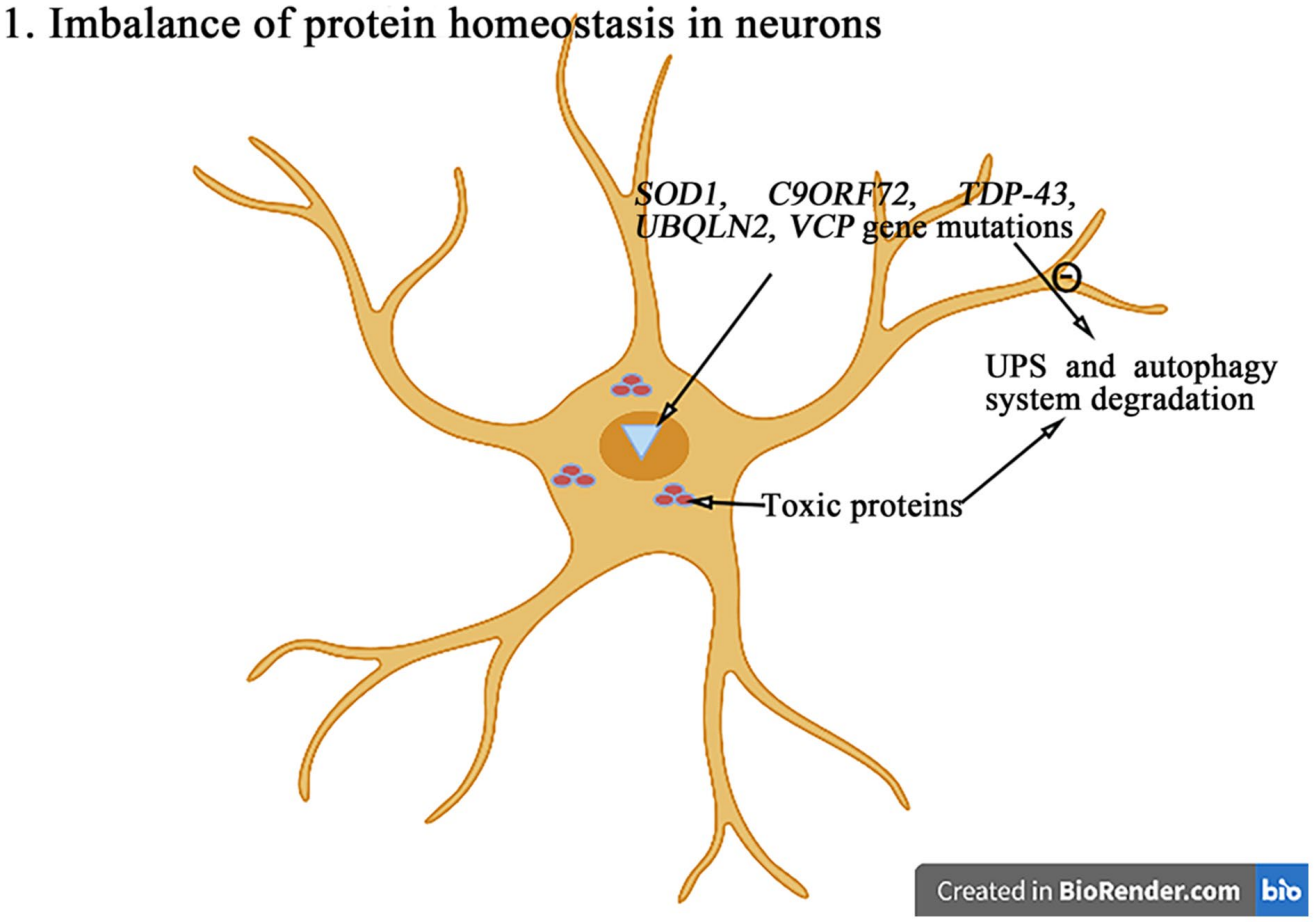
The mutations in genes such as *SOD1*, *C9orf72*, *TDP-43*, *ubiquilin-2*, and *VCP* can interfere with the UPS and autophagy degradation pathways, leading to the abnormal accumulation of toxic proteins and then leading to the protein homeostasis imbalance and ultimately inducing the cell death.

The maintenance of protein homeostasis is essential for cells to perform theirs functions (Figure 2). The protein imbalance can lead to the accumulation of misfolded proteins and ultimately cell death. Compared with other cell types, neurons are particularly susceptible to protein balance dysfunction (Kundra et al., 2020). The abnormal proteins accumulate excessively in neurons to form the inclusion bodies with the different characteristics and play the neurotoxic roles, resulting in the neuronal degeneration and necrosis. The degradation of abnormal proteins was mainly degraded through the intracellular protein UPS and the autophagy system. So far, more than 50 genes have been found to be associated with the pathogenesis of ALS (Li et al., 2023). And the degradation of abnormal proteins was mainly through the pathways of intracellular protein UPS and autophagy system degradation. Among more than 50 genes related to ALS, a variety of genes (*SOD1*, *C9orf72*, *TDP-43*, *ubiquilin-2*, *VCP*, etc.) directly affect the pathways of UPS and autophagy system (Nassif et al., 2017; Li et al., 2023).

### 2.1. *SOD1* gene mutation

A dominant missense mutation in the gene encoding *SOD1* was the first to be found to cause ALS, accounting for approximate 20% of all cases of familial ALS, in which the signature pathological feature is an insoluble *SOD1* ubiquitin-positive inclusion body within motor neurons. The chaperones molecules are the facilitators of protein folding and synthesis. The chaperon-assisted proteasome degradation is a system that facilitates the degradation of abnormal proteins by bridging misfolded and aggregated proteins to heat shock protein 70 (HSP70). This system participates in maintaining the protein homeostasis and promotes the refolding or removal of potentially toxic proteins (Tedesco et al., 2022). It has been found that heat shock proteins are encapsulated in the SOD1-positive aggregates, which reduces their ability to process the misfolded proteins in ALS (Farrawell and Yerbury, 2021). In addition, these abnormally aggregated proteins induce the endoplasmic reticulum (ER) stress and activate UPS to generate a vicious circle of abnormal toxic proteins production. Autophagy also appears to play an important role in the toxicity of mutant *SOD1*, which may be mediated by the removal of abnormal aggregates by HSP70 and its common partner B-cell lymphoma-2 associated athanogene 5 (Tedesco et al., 2023). However, a comparison between neuroblastoma × mouse spinal cord motor neuron cell line (NSC-34) motor neuron cell lines and C2C12 muscle cell lines showed that the activation efficiency of autophagy system was reduced in NSC-34 cells in the presence of *SOD1* mutant expression (Maurel et al., 2018). This further leads to excessive accumulation of mutated *SOD1*, creating a vicious cycle that leads to cell death.

### 2.2. *C9orf72* gene repeat mutation

In 2011, it was discovered that the most common genetic cause of ALS is the expansion of GGGGCC (G4C2) nucleotide repeats in an intron of *C9orf72* gene. Healthy individuals have less than 30 GGGGCC repeats, but in ALS patients, GGGGCC sequences can be amplified hundreds or thousands of times. The *C9orf72* mutation appears to drive the pathogenesis of ALS through the loss of normal function of *C9orf72* and the acquisition of toxic effects caused by the repeated amplification (Vant Spijker and Almeida, 2023), which leads to the RNA amplification, the dipeptide repeat proteins aggregation and the *C9orf72* haploid dysfunction. The RNAs containing (GGGGCC)n and the translation product the dipeptide repeat protein (GGGGCC)n are cytotoxic and can interfere with UPS and affect the protein degradation. ALS patients with *C9orf72* mutations exhibit the significant UPS impairment, and their characteristic pathological features are the ubiquitin-positive neuronal inclusions (Chua et al., 2022). The proteasome inhibitors selectively induce the death of motor neurons. In the *C9orf72* mutant, the proteasome subunit is significantly reduced, and the abnormal proteasome is presented in the inclusion body, suggesting that the *C9orf72* mutation promotes the occurrence and development of ALS by interfering with UPS. In addition, some studies have proposed the adverse effect of the down-regulation of *C9orf72* on the initiation of autophagy (Cali et al., 2019; Leskelä et al., 2019; Shi et al., 2019). The absence of *C9orf72* leads to the formation of light chain 3 (LC3)-positive autophagosomes and interferes with the autophagy process (Chua et al., 2022). In addition, *C9orf72* forms the complexes with Smith-Magenis syndrome chromosomal region candidate gene 8 and WD repeat-containing protein 41 and has the guanine nucleotide exchange factors activity against small guanosine triphosphateases (GTPase) (Beckers et al., 2021), which promotes guanosine triphosphate (GTP), the interconversion of GTP-Guanosine diphosphate (GDP) alters the configuration of Rab-GTPase and is involved in the formation of autophagosomes. The poor haploid function of *C9orf72* negatively affects several vesicular transport pathways (Rab1A, mannose-6-phosphate receptors (M6PRs), Rab5) and further affects the Rab protein activity and autophagy. The proteins encoded by *C9orf72* also interact with the Rab1A and unc-51-like autophagy activating kinase 1 complex to regulate the initiation of autophagy (Burk and Pasterkamp, 2019). Therefore, the *C9orf72* gene repeat mutation might contribute to the imbalance of protein homeostasis in neurons by the degradation decrease of abnormal toxic proteins due to the dysfunction of autophagy.

### 2.3. *TDP-43* gene mutation

The neurocytotoxicity induced by interacting the *TDP-43* gene nucleotide mutation is now widely recognized as a major pathological factor in ALS. TDP-43 is a nuclear protein encoded by *TDP-43* gene and involved in a variety of cellular functions, including gene transcription and RNA processing (Klim et al., 2021). The cytoplasmic TDP-43 may be involved in the accumulation of misfolded proteins in brain by inhibiting the UPS activity. The TDP-43 deposition has also been implicated in other neurodegenerative diseases, such as frontotemporal degeneration and Alzheimer’s disease. The recent studies (Yin et al., 2021) have shown that the TDP-43-induced neurotoxicity is associated with the damage of UPS. Its interaction with proteasome assemblers proteasome assembly chaperone 2 and postsynaptic density protein13 leads to impaired proteasome activity, while histone deacetylase 6 regulates TDP-43-induced the UPS damage through the autophagolysosome pathway (Lee et al., 2020). However, the molecular mechanism of TDP-43-induced neurotoxicity is largely unknown. Some studies (Lee et al., 2020) found that it may be related to the protein tyrosine kinase 2-TRAF family member associated NFkappaB activator (TANK)-binding kinase 1-sequestosome 1 axis, which regulates the UPS damage-induced neurotoxicity. Although the exact mechanisms about how to the *TDP-43* gene mutation effects the abnormal protein metabolizing have not been understood up to now. According to the current studies, the mutations in *TDP-43* gene may disrupt the protein homeostasis and impair the function of UPS and autophagy, which are essential for protein degradation.

### 2.4. *Ubiquilin-2* gene mutation

Ubiquilin-2 is a protein encoded by *ubiquilin-2* gene. It belongs to the ubiquilin family of proteins that have a ubiquitin-like domain at N-terminus and a ubiquitin-associated domain at C-terminus. Ubiquilin-2 binds to various ubiquitinated substrates through its C-terminal domain and directs them to UPS for the protein degradation through its N-terminal domain. Therefore, the main function of ubiquilin-2 is to transport the ubiquitinated target proteins to proteasome for degradation. Moreover, ubiquilin-2 can also function as an autophagy receptor by directly binding to microtubule-associated protein LC3, a marker of autophagosomes, through its ubiquitin-associated domain, which regulates the activity of the mechanistic target of rapamycin complex 1 and the acidification of lysosomes, and thus modulates the autophagy maturation13 (Wu et al., 2020). In the related experiments, the mutant *ubiquilin-2* gene disrupts the protein homeostasis. The overexpression of this gene resulted in the motor neuron loss and the accumulation of autophagy substrates, including both sequestosome 1 and LC3-II. These effects reduce the autophagy flux and impaire the autophagic acidification (Wu et al., 2020). Moreover, the mutations in *ubiquilin-2* gene weaken the interaction between ubiquilin-2 protein and HSP70, reducing the ability of UPS to clear the abnormally aggregated proteins, which in turn results in the abnormal protein aggregation (Zhang et al., 2021). Therefore, the ubiquilin-2 protein plays an important role in the regulation of neuronal protein homeostasis.

### 2.5. *VCP* gene mutation

*VCP* gene encodes an enzyme called VCP, which belongs to the ATPase family AAA domain (ATPases associated with diverse cellular activities) family of proteins. VCP can bind to the ubiquitinated target proteins and participate in their degradation through the ubiquitin-proteasome pathway. One of VCP functions is to maintain the proteins solubility or reverse the aggregation of insoluble, misfolded proteins before they are delivered to proteasome. The most characteristic function of VCP is to participate in the ER-associated protein degradation, a system related to UPS (Wani and Weihl, 2021). According to a recent study (Ziff et al., 2022), the *VCP* mutation can impair the maturation and lysosomal *FUS*ion of autophagosomes. Motor neurons and astrocytes derive from the induced pluripotent stem cells with the pathogenic *VCP* mutations exhibit the abnormal cytoplasmic accumulation of TDP-43 protein, ER stress, mitochondrial dysfunction and oxidative stress, leading to cell death. The presence of these mutant protein aggregates is thought to impair the proteasome and autophagy degradation pathways and may be a key mediator of protein homeostasis imbalance in the pathogenesis of ALS.

### 2.6. Other ALS-related genes mutations

The mutations in *TANK-binding kinase 1* (*TBK1*) gene may impair the clearance of proteins and the turnover of mitochondria, resulting in the neuronal damage and the activation of toxic protein responses. In ALS patients, the mutations in the vesicle-associated membrane protein-associated protein B (VAPB) gene induce ER stress and impair UPS, leading to the protein homeostasis reduction and the motor neuron loss. The optineurin gene regulates apoptosis by binding to the linear ubiquitin chains. In summary, the nucleotide variations in the ALS-related genes mainly affect the protein homeostasis through the ubiquitin-proteasome pathway and promote the apoptosis and autophagy pathway through impairing the mitochondria functions, which are involved in the molecular genetic pathogenesis of ALS (Maurel et al., 2018).

## 3. RNA metabolism disorder

**Figure 3:**
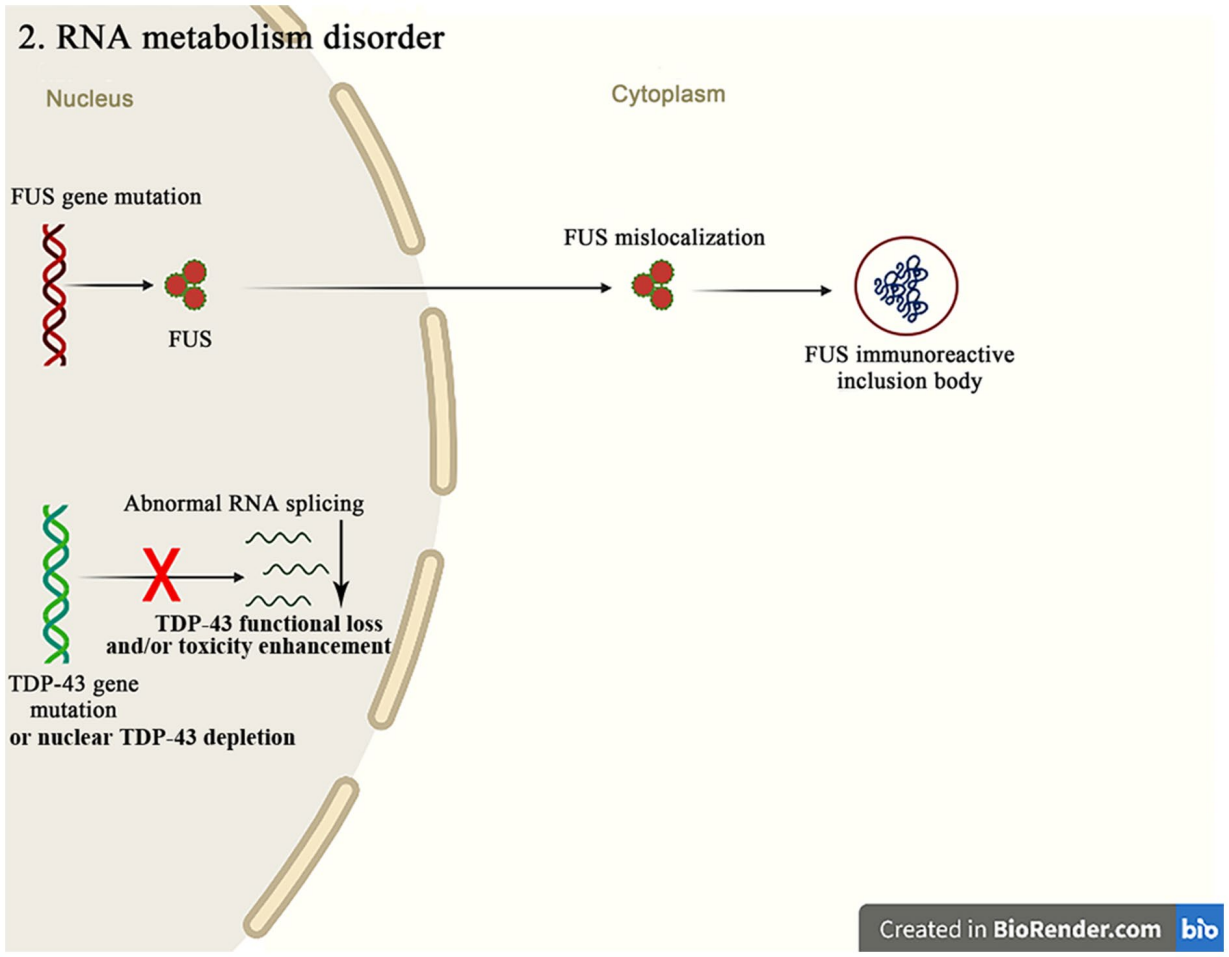
The *FUS* mutations mainly cause the mislocalization of *FUS* in cytoplasm, resulting in the *FUS* immunoreactive inclusion bodies. The abnormal RNA splicing pathway caused by the *TDP-43* mutation or the nuclear *TDP-43* depletion promotes the pathways of ALS occurrence through the TDP-43 functional loss and/or toxicity enhancement.

The molecular genetic pathogenesis of ALS is advancing with the recent discovery of many gene mutations in ALS patients, many of which encode RBPs, such as *TDP-43*, *FUS*, *ataxin 2*, TATA box binding protein-associated factor 15 (*TAF15*), ewing sarcoma breakpoint region 1 (*EWSR1*), heterogeneous nuclear ribonucleoprotein A1 (*hnRNPA1*), hnRNPA2/B1, matrin3 (*MATR3*) and T cell-restricted intracellular antigen-1 (*TIA-1*) (Figure 3). RBPs are an evolutionarily conserved group of proteins that participate in multiple steps of RNA metabolism, such as splicing, polyadenylation, mRNA stability, localization and translation (Xue et al., 2020). *TDP-43* and *FUS* are the major pathogenic genes in familial ALS. Normally, these proteins localize to nucleus and are involved in transcription, splicing, non-coding RNA metabolism, and micro-RNA biogenesis (Mackenzie et al., 2017). Although these proteins are mainly confined to nucleus, they can be mislocalized to cytoplasm in the postmortem brain tissue of mutation carriers. This nuclear depletion of RBPs may be harmful and cause the severe transcriptome abnormalities, while the cytoplasmic aggregates may also induce toxicity. These findings highlight the important role of mutations in the different RBPs genes in the pathogenesis of ALS (Humphrey et al., 2020).

### 3.1. *FUS* gene mutations

FUS protein is an RBPs that participates in the RNA metabolism and the DNA repair. It is mainly localized in nucleus and regulates transport, the local translation and stability of RNA. Specifically, FUS also binds to the RNA of many receptors and transporters involved in the glutamatergic and GABAergic signaling (Sahadevan et al., 2021). The *FUS* mutations predominantly affect the 3′ arginine/glycine-rich regions and the nuclear localization signal domains and are mostly missense. These mutations mainly lead to the FUS mislocalization in cytoplasm, which leads to the production of FUS immunoreactive inclusion bodies leading to the neurons degeneration in the pathogenesis of ALS. The mutant FUS toxicity may result from the irreversible formation of cytoplasmic inclusion bodies or the detergent-resistant protein aggregates, which impair the RNA processing (Humphrey et al., 2020). Therefore, both the loss of nuclear function and the increased cytotoxicity of FUS contribute to the pathogenesis of ALS. *In vivo* studies in mice have shown that the disease-related gene mutations in *FUS* alter the properties of proteins, leading to the abnormal phase transition and loss and toxic gain of function, and induce the conformational changes in the related proteins, resulting in one of the pathological markers of ALS, which is the magnocellular-like aggregation, and the further consolidation and growth of fibrous structures (Korobeynikov et al., 2022). Therefore, the *FUS* mutations may partly explain the molecular genetic pathogenesis of ALS.

### 3.2. *TDP-43* gene mutation

TDP-43 is a RBPs encoded by *TARDBP* gene. It contains two RNA recognition motifs, a nuclear export signal and a glycine-rich region at C-terminal. TDP-43 regulates various aspects of RNA metabolism, such as transcription, the alternative splicing and the mRNA stability. It also participates in the diverse cellular processes, such as apoptosis, cell division and axon transport. TDP-43 regulates the mRNA expression of many proteins involved in the development, survival and synaptic transmission of neurons, as well as neuroplasticity in the central nervous system (CNS) (Ling, 2018). The recent studies have suggested that the TDP-43 toxicity in the molecular pathogenesis of ALS may be related to both loss of function (due to nuclear depletion of TDP-43) and gain of toxic function (due to cytoplasmic aggregation of TDP-43) (Deshaies et al., 2018). This mechanism suggests that the abnormal RNA splicing induced by the *TDP-43* mutations or nuclear depletion of *TDP-43* contributes to the pathogenic of ALS through the loss of function and/or gain of toxicity. Moreover, TDP-43 has been shown to act as a specific translation enhancer for several mRNAs, including DENN/MADD domain containing 4A (*Dennd4a*) and calmodulin binding transcription activator 1 (*Camta1*), which are implicated in the neurodegeneration of ALS. The *A315T* mutation in *TDP-43* enhances the direct binding and translation of these mRNAs, leading to increase the ALS susceptibility (Neelagandan et al., 2019).

### 3.3. Other ALS-related genes mutations

Angiotensinogen (ANG) is a stress-induced secreted ribonuclease with both nuclear and cytoplasmic activities. In the cell nucleus, ANG promotes the transcription of ribosomal RNA (Prehn and Jirström, 2020). It has found that the mutations in *ANG* gene disrupt the formation of RNA molecules, leading to the accumulation of toxic RNA fragments and the reduction of protein levels in motor neurons, and contributing to the development of ALS. Furthermore, ANG can promote the survival of motor neurons and prevent their degeneration, suggesting that restoring ANG levels may be a therapeutic strategy for treating ALS (Aluri et al., 2020). Overall, although the exact mechanism linking ANG and ALS is still being studied, the current research suggests that ANG may play a key role in the pathogenesis of ALS. The mutations in both hnRNPA2B1 and hnRNPA1 genes are the rare causes of both ALS and multisystem proteinopathy. Both hnRNPA1 and hnRNPA2/B1 have the similar functions in regulating the mRNA maturation, splicing, translation and stability, but they play the different roles in the transcription regulation (Kim and Taylor, 2017). HnRNPA1 is a part of RBPs complex that assembles with RNA to form ribonucleoproteins. HnRNPs are concentrated in the cell nucleus and play a role in regulating the pre-mRNA splicing, mRNA stability, transcription and translation (Beijer et al., 2021). HnRNPA2B1 is a protein involved in the RNA processing and transporting within the cell nucleus. It has found that mutations in both hnRNPA2B1 and hnRNPA1 genes lead to the abnormal accumulation of protein in motor neurons, disrupting the RNA processing and ultimately leading to the development of ALS. The knockdown of hnRNPA2/B1 also disrupts the alternative splicing and leads to impair the cognitive function (Baradaran-Heravi et al., 2020). T-cell intracellular antigen-1 (TIA-1) is a protein with multiple functions in the RNA metabolism, including the mRNA splicing, the translation inhibition and the mRNA silencing. Several missense mutations in TIA-1 gene have been identified as being associated with the pathogenesis of ALS. The TIA-1 mutations can alter the ability of protein to undergo the liquid–liquid phase separation and can damage the disassembly of stress granules (SGs) *in vitro*. It suggests that the TIA-1 mutations may be involved in the development of neurodegenerative diseases such as ALS (Mackenzie et al., 2017). TIA-1 can bind to the RNA molecules and form SGs in motor neurons. In ALS patients, these SGs have been found to contain the aggregated proteins and the RNA fragments, which can lead to the dysfunction and degeneration of neurons (Sekiyama et al., 2022). Ataxin-2 (ATXN2) is a RBPs that belongs to the like-Smith family and is involved in the regulation of RNA metabolism. ATXN2 plays a key role in regulating the toxicity of TDP-43 and FUS through the direct protein–protein interactions, and the reducing the levels of ATXN2 has been shown to inhibit TDP-43-mediated neurotoxicity (Becker et al., 2017). As the above mention, the gene mutations encoding many RBPs are highly associated with the pathogenesis of ALS. In addition, the dysregulation of RBPs also significantly contributes to the pathogenic mechanisms of ALS due to the impaired nuclear-cytoplasmic transport, the post-translational modifications, aggregation and the abnormal RNA sequestration.

## 4. Mitochondrial dysfunction

**Figure 4:**
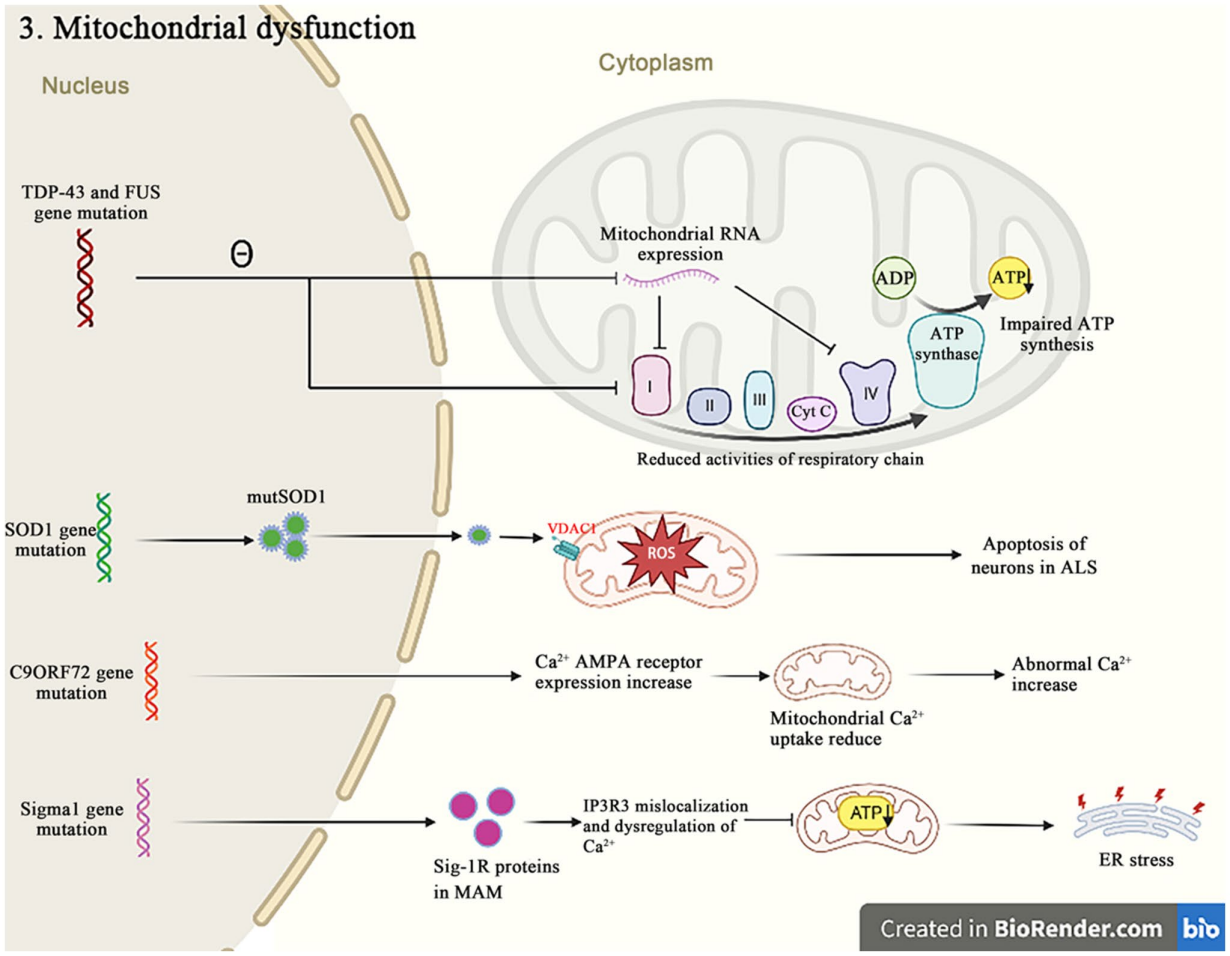
Both *TDP-43* and *FUS* mutant isoforms, respectively, disrupt the mitochondrial RNA expression and interact with the mitochondrial ATP synthase catalytic subunits, resulting in the abnormal mitochondrial membrane potential, and reducing the ATP production and the oxygen consumption. The *SOD1* mutations impair the ability of mitochondria to eliminate ROS. The *C9orf72* mutations increase the concentration of neuronal Ca^2+^ influx and the overexpression of polyGR that causes the mitochondrial dysfunction by altering protein binding patterns on mitochondria. The Sigma-1R mutants cannot bind to IP3R, leading to the Ca^2+^ imbalance in MAM and exacerbating the related pathways of ER stress-induced neuronal death.

Besides their canonical roles as the energy producers, mitochondria are also implicated in other cellular dysfunctions that contribute to the pathogenic of ALS, such as the Ca^2+^ homeostasis, the apoptosis regulation and the protein quality control (Figure 4). A recent study reported that mutations in 16 ALS pathogenic genes compromised the integrity of mitochondria-associated membranes and impaired the mitochondrial function (Sakai et al., 2021). The impaired mitochondrial electron transport chain function in various neurodegenerative diseases including ALS affects the oxygen consumption and the membrane potential, and causes to increase reactive oxygen species (ROS) and decrease the adenosine triphosphate (ATP) production. The excessive ROS induce the oxidative stress in cells and accelerate the neuronal degeneration and death (Delic et al., 2018). These evidences strongly suggest that the dysfunction of mitochondria is critical in the development of ALS.

### 4.1. *TDP-43* gene mutation

**Figure 5:**
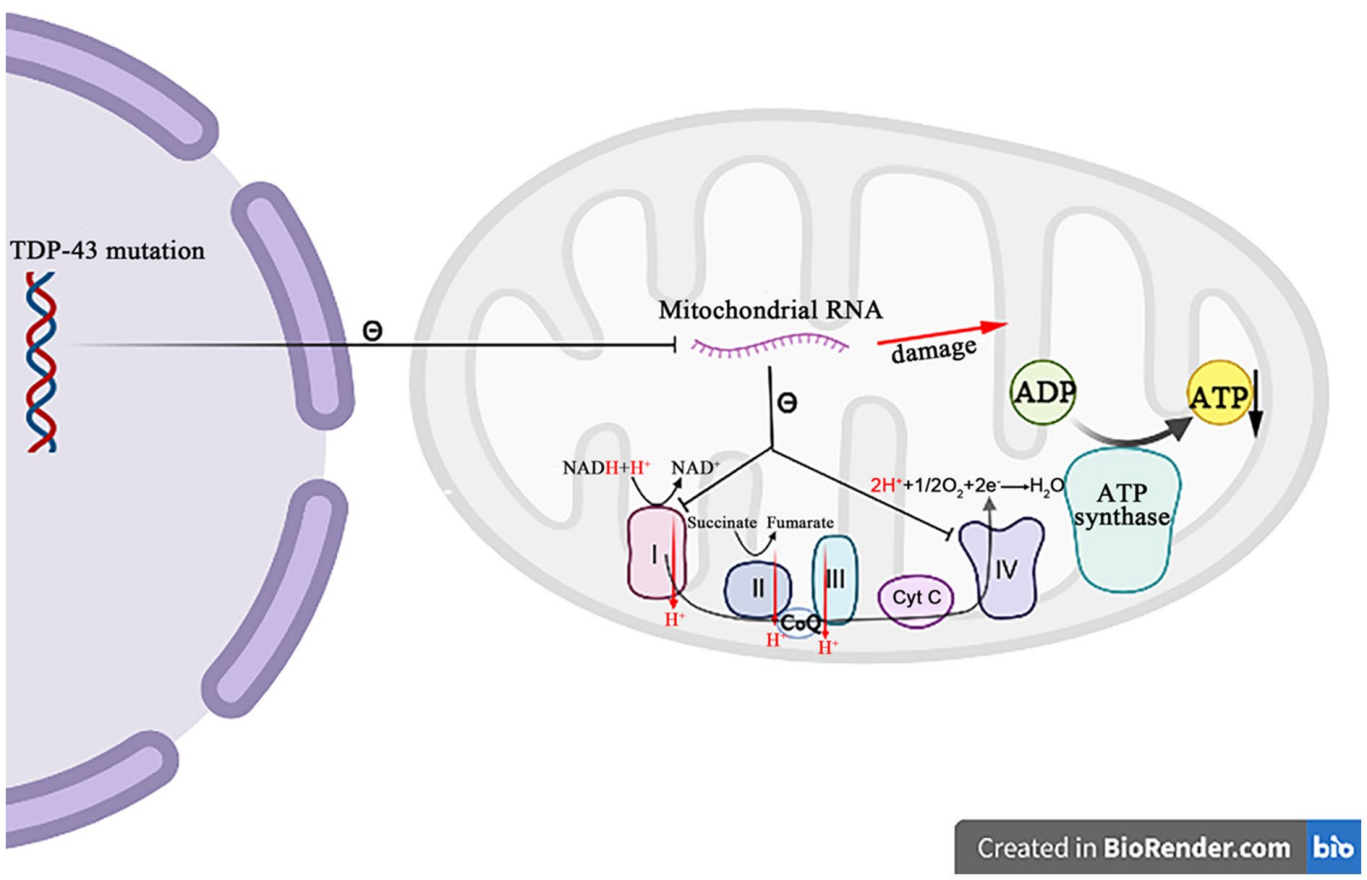
The mutation *TDP-43* inhibits the mitochondrial RNA expression, resulting in the mitochondrial crest damage, decreasing the respiratory chain complex I and IV activity, and decreasing the ATP synthesis.

A growing body of evidence suggests that *TDP-43* is closely related to the mitochondrial dysfunctions. When *TDP-43* is mutated, the protein levels of respiratory chain complex I subunit NADH phanquinone redox enzymes 3 and 6 are affected, thereby impinging the assembly and function of complex I, resulting in the abnormal mitochondrial endometrial potential, decreasing the ATP production and the oxygen consumption (Wang et al., 2017). The *TDP-43* mutation affects not only nucleus, but also mitochondria, where it suppresses the expression of mitochondrial RNA, which leads to the mitochondrial ridge damage, reduces the activities of respiratory chain complex I and IV, and impairs the ATP synthesis (Figure 5) (Shenouda et al., 2018; Wang et al., 2019). It has also been recently reported that the abrupt overexpression of *TDP-43* triggers a unique self-destructive pathway of mitophagy, leading to the mitochondrial death (Gautam et al., 2019). Therefore, the *TDP-43* gene nucleotide mutations participate in the pathogenesis of ALS through destroying the function and construct of mitochondria.

### 4.2. *FUS* gene mutation

Similar to *TDP-43*, the *FUS* mutants cause the mitochondrial morphological and functional abnormalities, which has been shown that the FUS mutants can affect the mitochondrial function by disrupting the transcription of mitochondrial complex required for the ATP production. Within mitochondria, the protein encoded by the mutant *FUS* interacts with the mitochondrial ATP synthase catalytic subunit ATP5B, disrupting the assembly of ATP synthase complexes and reducing the mitochondrial ATP synthase activity, thereby reducing the mitochondrial ATP synthesis, inducing the mitochondrial ridge loss, and increasing the mitochondrial fragmentation (Deng et al., 2018). The *FUS* mutations were further found to be associated with the disruption of ER-mitochondrial association, and FUS was shown to activate glycogen synthase kinase-3beta, a regulator of ER-mitochondrial association, which inhibits the interactions between VAPB and protein tyrosine phosphatase interacting protein 51, resulting in the disruptions in cellular Ca^2+^ homeostasis and the mitochondrial production defect of ATP (Sakai et al., 2021). Overall, there is the substantial evidence that the *FUS* gene mutations alter the mitochondrial dynamics, functions and structure in the pathogenesis of ALS.

### 4.3. *SOD1* gene mutation

The protein encoded by *SOD1* is a ubiquitous homologous dimer enzyme involved in the primary antioxidant defense of cells. The mutations in SOD1 (mutSOD1) aberrantly target the protein to the mitochondrial intermembrane space, impairing the ability of mitochondria to eliminate ROS and causing the mitochondrial damage. There are the growing evidences that the abnormal binding of mutSOD1 protein to voltage-dependent anion channel-1 (VDAC1) contributes to the progression of ALS (Shoshan-Barmatz et al., 2020). VDAC1 is an integrin of mitochondrial outer membrane that controls the metabolite flow, allows the adenosine nucleotide transport, and regulates the cellular processes such as apoptosis and the Ca^2+^ homeostasis. mutSOD1 specifically binds to the N-terminal domain of VDAC1, reducing its channel conductance and leading to the breakdown of mitochondrial function. In addition, it has been found that the mutSOD1 aggregates interact with the mitophagy receptors in neuronal cells, inhibiting their function as the mitochondrial flux promoters (Semmler et al., 2020). The mutations in *SOD1* gene also inhibit the ATP synthesis and further inhibit the function of sodium–potassium pump, thereby impacting the electrical activity of neurons. The *SOD1* gene mutations impair the mitochondrial membrane and its associated structures, leading to the imbalance of sodium–potassium and Ca^2+^ ion homeostasis, the dysfunction of ROS metabolism and autophagy, and ultimately increased the apoptosis of neurons in the pathogenesis of ALS.

### 4.4. *C9orf72* gene repeat mutation

In 2016, Benson et al. first reported the correlation between *C9orf72*-ALS mutation and mitochondrial dysfunction (Benson et al., 2021). The mechanism of mitochondrial dysfunction caused by the *C9orf72*-ALS mutation has been extensively studied. The current evidences suggest that it is mainly related to Ca^2+^ dysregulation and the overexpression of polyglycine-arginine (polyGR) dipeptides. In patients with *C9orf72*-ALS, the influx of Ca^2+^ into nerve cells is increased, the recovery time of intracellular Ca^2+^ is prolonged, and the buffering capacity of cytoplasm for Ca^2+^ is reduced (Candelise et al., 2022). It was found that this may be caused by increasing the expression of Ca^2+^AMPA receptor and reducing the production of mitochondrial Ca^2+^ uptake 1 and 2 Ca^2+^ channels, leading to the decreased uptake capacity of Ca^2+^ by mitochondria, which causes the abnormal increase of Ca^2+^ concentration in cytoplasm and causing the damage to cells. The overexpressed polyGR can lead to the mitochondrial dysfunction by changing the binding mode of proteins on mitochondria (Benson et al., 2021). Another possibility is that polyGR binds to the ATP5A1 subunit of mitochondrial complex line V and induces its degradation through ubiquitination. Such damage inhibits the redox reactions in mitochondria and leads to the lack of cell energy. The lack of energy in cell leads to a series of irreversible damage to neurons in the pathogenesis of ALS.

### 4.5. *Sigma-1R* gene mutation

The recent studies have shown that *Sigma-1* gene is involved in the pathogenesis of ALS. *Sigma-1* gene encodes Sigma-1R protein, a molecular chaperone located in mitochondria-associated endoplasmic reticulum membranes (MAM). Sigma-1R can bind to inositol 1,4,5-trisphosphate receptor (IP3R) and facilitate Ca^2+^ flux into mitochondria, regulating Ca^2+^ homeostasis in the neuronal cytoplasm (Smith et al., 2019). The Sigma-1R mutants fail to bind IP3R, resulting in the mislocalization of IP3R3 and the dysregulation of Ca^2+^ in MAM. The disorders of MAM and Ca^2+^-related homeostasis affect the mitochondrial function and the neuronal survival (Wu et al., 2021). Moreover, it has shown that familial ALS patients exhibit a missense or non-sense mutation in the code of *Sigma-1R*, which replaces glutamine in the glutamate position. The expression of Sigma-1R protein in this mutant reduces the mitochondrial ATP production, inhibits the proteasome activity and leads to the mitochondrial damage, thus exacerbates the ER stress-induced neuronal death in the pathogenesis of ALS.

### 4.6. Other ALS-related genes mutations

The mitochondrial homolog protein coiled-coil-helix-coiled-coil-helix domain containing 10 (CHCHD10) can form approximate 220 kDa complexes in the mitochondrial intermembrane space and cooperate to regulate the mitochondrial function (Burstein et al., 2018; Straub et al., 2018). It is reported that CHCHD10 regulates the mitochondrial cytochrome C oxidase activity and respiration during hypoxia (Purandare et al., 2018), and many studies suggest that CHCHD10 may play a role in the mitochondrial dynamics and cristae organization. It has been found that the mutations in *CHCHD10* gene are associated with defects in the mitochondrial dynamics and cristae, leading to the accumulation of toxic waste and ultimately damaging motor neurons, resulting in the pathogenesis of ALS. The recent studies on the adolescent ALS have also found that the mutations in *C19ORF12* gene can lead to a form of ALS called ALS10, which is characterized by weakness and atrophy in the lower limb muscles, as well as spasms and difficulty in movement (Kim et al., 2016). The mutations in *C19ORF12* gene are rare, accounting for only a small fraction of all ALS cases. C19orf12 is a protein that localizes to both mitochondria and ER. The mutations of this protein can disrupt the lipid homeostasis in mitochondria and lead to the iron deposition in cells (Khani et al., 2019).

## 5. Glutamate-mediated excitatory toxicity

**Figure 6:**
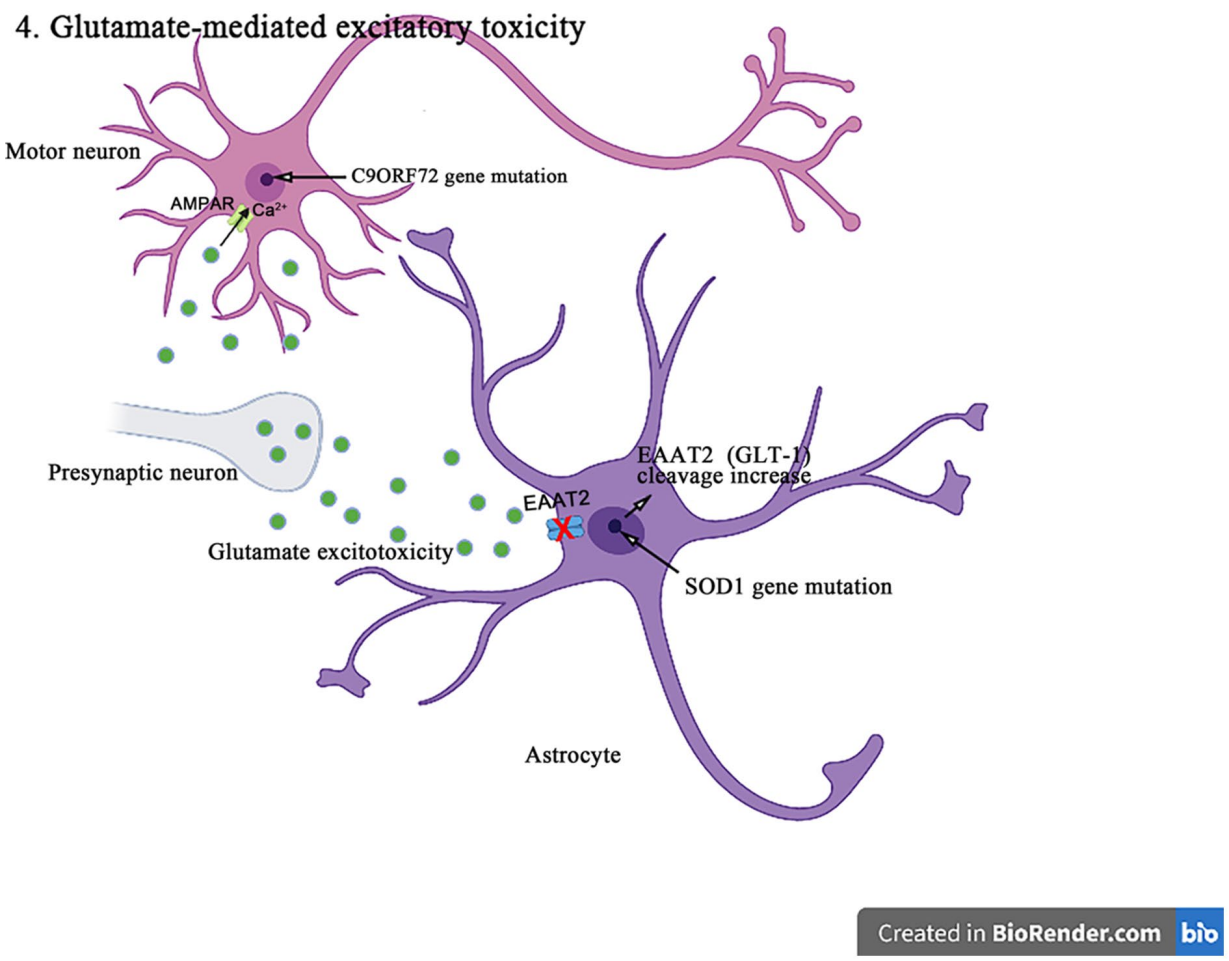
The *SOD1* mutants enhance the caspase-3-mediated cleavage of EAAT2 (GLT-1), resulting in impairing the glutamate uptake and producing excitotoxicity in the pathogenesis of ALS. In addition, *C9orf72* mutations lead to the production of toxic dipeptide repeat sequences, increasing the expression and Ca^2+^ permeability of GluA1, leading to excitotoxicity in motor neurons in the pathogenesis of ALS.

The accumulation of excitatory mediators and neuronal damage, a mechanism known as excitotoxicity, can result from the overactivation of glutamate receptors, the failure to clear the glutamate neurotransmitters from the synaptic cleft, or the increased post-synaptic sensitivity to glutamate (Olloquequi et al., 2018) (Figure 6). Glutamate is the main excitatory neurotransmitter in CNS, initiates a rapid signal transmission in synapses and then reuptake into the surrounding glial cells, especially astrocytes. GLAST and GLT-1 and their homologous EAAT1 and EAAT2 of astrocytes are major transporters that absorb the synaptic glutamate to maintain the optimal extracellular glutamate levels, thus, prevents the accumulation of glutamate in the synaptic cleft and the consequent excitatory toxicity. Therefore, the dysregulation of glutamate transporters in glial cells may play an important role in the excitatory toxicity and the related neuropathogenesis (Pajarillo et al., 2019). In addition, glutamate can induce the cytoplasmic Ca^2+^ increase, and the intracellular Ca^2+^ influx and accumulation are key factors causing the excitatory toxicity. Alpha-amino-3-hydroxy-5-methyl-4-isoxazole propionic acid-type glutamate receptors (AMPAR) is a key subtype of ionic glutamate receptors that mediates the excitatory synaptic transmission in CNS and peripheral nervous system. AMPAR is an integrated transmembrane protein assembled by AMPA selective glutamate receptor (GluA1-4) subunit (also known as GRIA1-4 or GluR1-4) into a tetramer complex, in which the presence of GluA2 subunit in AMPAR prevents the influx of cations (such as Ca^2+^) and affects the ion permeability in neurons (Cull-Candy and Farrant, 2021).

### 5.1. *SOD1* gene mutation

The increasing evidences suggest that the abnormal posttranslational modification is associated with the pathogenesis of EAAT1 (GLAST)/EAAT2 (GLT-1) dysfunction. EAAT2 (GLT-1) has a caspase-3 consensus sequence and can be activated *in vitro* by the caspase-3 cleavage. It has been shown that the *SOD1* mutant mice enhance the caspase 3-mediated EAAT2 (GLT-1) cleavage, leading to the formation of truncated EAAT2 (GLT-1) (sumoylated EAAT2 C-terminus fragment, CTE-SUMO1). The cleavage site mutation does not affect the EAAT2 (GLT-1) activity in ALS mice, but delays the disease progression and prolongs lifespan (Rosenblum et al., 2017). In addition, the accumulation of CTE-SUMO1 in astrocytes increases toxicity, leading to the impaired growth of neurons and axons (Hirschberg et al., 2021). These findings suggest that the abnormal GLT-1 cutting impairs the glutamate uptake and contributes to the excitatory toxicity in the pathogenesis of ALS. Moreover, it was found that the histone deacetylase (HDAC) inhibitors such as a selective inhibitor of class II HDAC (MC1568), valproic acid, and sodium butyrate increased the EAAT1 (GLAST)/EAAT2 (GLT-1) expression and the glutamate uptake in both *in vitro* and *in vivo* models of ALS and Mn-induced neurotoxicity (Johnson et al., 2018). MC1568 can also regulate the EAAT2 transcription, improve the EAAT2 expression and the post-translational modification, and has been proven to have the therapeutic effects in the subclinical models of ALS (Lapucci et al., 2017).

### 5.2. *C9orf72* gene repeat mutation

Numerous studies have been conducted to understand the biological consequences of *C9orf72* repeated amplification, one of which is the production of toxic dipeptide repeats (Gendron and Petrucelli, 2018). The recent studies have also shown that the arginine-rich dipeptide repeats (polyglycine-arginine/proline-arginine (polyGR/PR)) is one of major neurotoxicity sources. In the drosophila-related experiments (Xu and Xu, 2018), the increased levels of extracellular glutamate and intracellular Ca^2+^ were detected in the ventral nerve cords of GR/PR-expressing larvae, accompanied by the significant increases in the synaptosomes and the active regions of larval neuromuscular junctions. The *C9orf72* mutation results in the increased expression of AMPAR subunit GluA1 accompanied by the increased permeability to Ca^2+^. The excessive Ca^2+^ load in neurons can also produce the significant cytotoxicity. The inhibiting expression of vesicular glutamate transporters in the presynaptic glutamatergic motor neurons or blocking N-methyl-d-aspartic acid receptor can effectively save the motor deficits induced by polyGR/PR and prolong lifespan, which thus indicates the mechanism of autonomic excitatory toxicity. In addition, the EAAT2 splicing may be influenced by C9orf72 dipeptide repeats and lead to the decreased uptake of glutamate by astrocytes and increased the excitatory toxicity of motor neurons (Shi et al., 2018).

### 5.3. Other ALS-related genes mutations

In addition, the *TDP-43* mutations downregulate adenosine deaminase acting on RNA editing enzyme adenosine deaminase acting on RNA 2 (ADAR2), leading to the failure of GluA2 glutamine/arginine (Q/R) site editing in *GluA2* pre-mRNA (Yamashita and Kwak, 2019), which results in the significant loss of motor neurons in most sporadic ALS patients.

## 6. Obstruction of material transport within neurons

**Figure 7:**
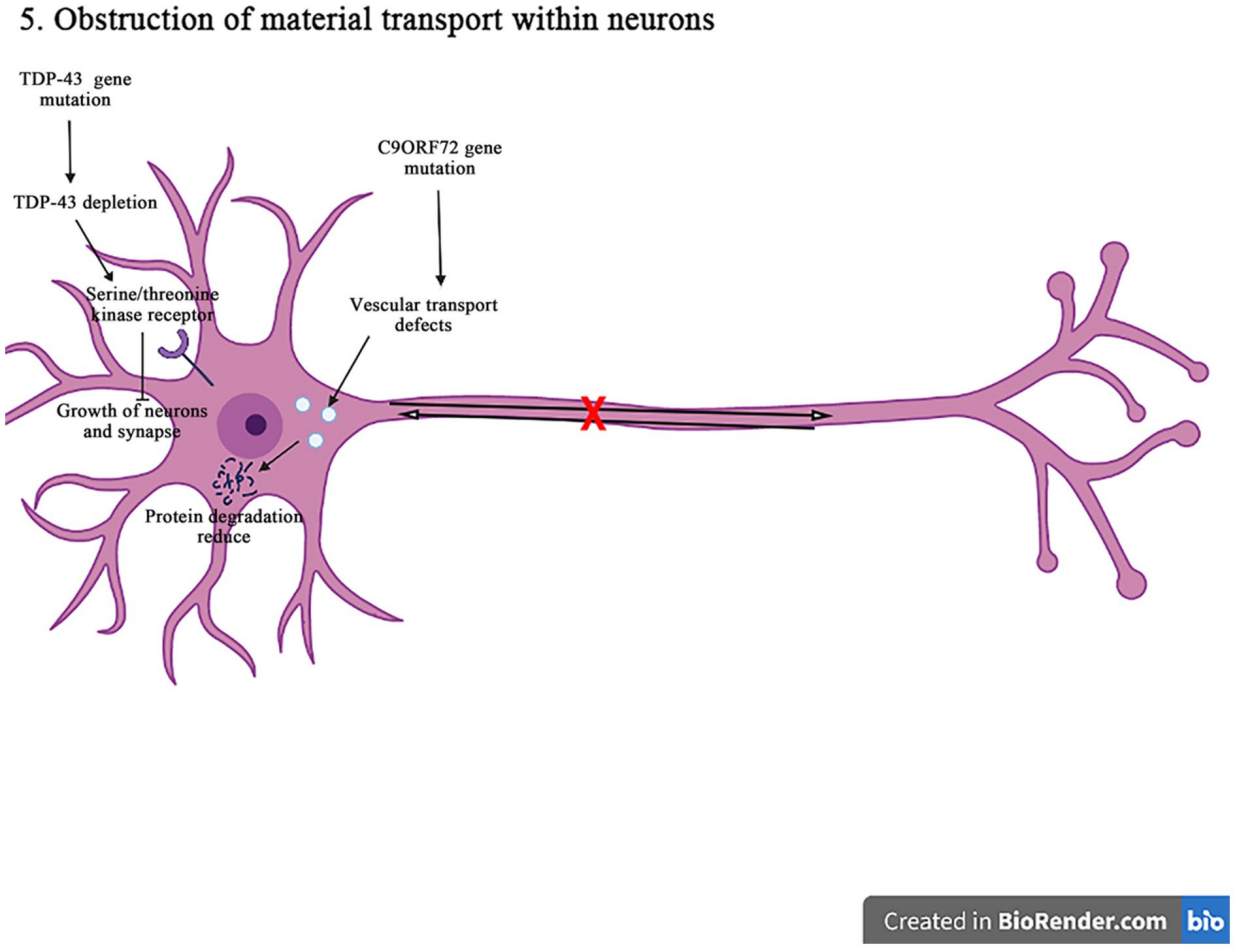
The *C9orf72* gene mutations can affect the vesicular transport pathway, reduce the protein degradation, and cause the neurodegeneration and death of neurons. The *TDP-43* gene deletion reduces the signaling pathways for the epidermal growth factor receptors and the serine/threonine kinase receptors, affecting the neuron survival and the axon innervation. In addition, it also interferences with the material circulation and target protein transport pathways on the neuronal cell membrane, ultimately damaging neurons.

Motor neurons are highly dependent on the axonal transport mechanisms to maintain the normal structure and function (Figure 7). It not only determines the nutrient supply, the energy metabolism, and the signal transduction of neuronal axons but also affects various physiological functions and the homeostasis of neurons. *C9orf72*, *TDP-43*, *FUS*, and other gene mutations are closely related to neuronal transport disorders in the pathogenesis of ALS.

### 6.1. *C9orf72* gene repeat mutation

The expansion of *C9orf72*GGGGCC nucleotide repeat can reduce the expression of *C9orf72*, and accumulate the RNA containing GGGGCC repeat in the nuclear foci. It can lead to the production of toxic dipeptide repeat protein, and ultimately impair the intracellular vesicle transport, resulting in decreasing the protein degradation and increasing aggregation, and inducing the neuronal degeneration and death in the pathogenesis of ALS. It has shown that in motor neurons, C9orf72 protein is localized to Rab5-positive endosomal vesicles, which can promote the role of Rab5 in vesicle transport and early endosomal maturation, assist Rab protein in GDP-GTP exchange, and improve the survival rate of neurons (Shi et al., 2018; Pang and Hu, 2021). The mutation C9orf72 inhibits the maturation of intracellular vesicles, reduces the production of lysosomes, and affects the protein degradation. In addition, the C9orf72 mutation can affect the transmembrane receptor M6PRs. It is found that the mutation C9orf72 can reduce the rate of aggregation and migration of M6PRs, which promotes the protein degradation by transporting materials to lysosomes. Therefore, the misshipment of M6PRs may lead to reduce the protein degradation in the pathogenesis of ALS (Starr and Sattler, 2018).

### 6.2. *TDP-43* gene mutation

The disruption of receptor transport in ALS is also associated with *TDP-43* gene. It is found that the knockout of *TDP-43* gene leads to epidermal growth factor receptor expressed in cell membrane surface is reduced, and epidermal growth factor and epidermal growth factor receptor signaling can promote the survival of neurons, mature and grow (Chou et al., 2018). When the epidermal growth factor receptor expression is reduced on the surface of cell membrane, the circulation of cell surface receptors is impaired, thus affecting the neuronal survival and the axonal innervation. Furthermore, the *TDP-43* depletion affects serine/threonine kinase receptor (bone morphogenetic proteins receptor). Bone morphogenetic proteins receptors can promote the synaptic growth through the phosphorylation of downstream mothers against decapentaplegic protein (a protein called Mad (mothers against decapentaplegic)), and the depletion of TDP-43 will lead to decrease bone morphogenetic proteins receptor signal transduction and ultimately affect the growth of neurons and synapses (Burk and Pasterkamp, 2019). The *TDP-43* gene mutation can affect the circulation of materials on the cell membrane of neurons and the transport of target proteins during the degradation process, thus damaging neurons in the pathogenesis of ALS.

### 6.3. Other ALS-related genes mutations

The SOD1 mutations gain an affinity for both actin and microtubule proteins, which impairs the neuronal growth and the mitochondrial fission (Osking et al., 2019; Muñoz-Lasso et al., 2020). The cytoplasmic inclusions of FUS prevent the ribonucleotide cargoes from reaching their normal destinations by abnormally sequestering the transport proteins and affecting the post-translational status of microtubule tracks (Yasuda et al., 2017). Kinesin family member 5A (KIF5A), a motor protein, drives the microtubule tracks to mediate the anterograde transport of mitochondria, vesicles and signaling molecules. KIF5A is expressed only in motor neurons, is critical to development and function of motor neurons, and is the only reported motor protein which mutations cause ALS (He et al., 2020; Baron et al., 2022). The reduction of KIF5A in zebrafish or rat hippocampal neurons leads to hyperexcitability, the axonal degradation and defects in the mitochondrial transport (Zhao et al., 2020). The profilin 1 (PFN1) variants (A20T, C71G, T109M, M114T, E117G, G118V, R136W, and Q139L) cause the autosomal dominant form of ALS by interfering with the actin dynamics in two opposite pathways. Firstly, PFN1 directly binds to the actin monomers, blocking the sites for new monomer addition, and thus preventing the actin filament assembly (Pimm et al., 2020; Liu et al., 2022). Secondly, PFN1 enhances the actin filament assembly by interacting with PFN, a profilamentous actin-binding protein (Schmidt et al., 2021). Taken together, these observations strongly suggest that PFN1 promotes ALS by regulating both microtubule tracks and transport machinery, such as motor proteins, dynein or spastin (Pinto-Costa et al., 2020). Therefore, understanding the underlying mechanisms of such regulation is indispensable for developing specific drug targets for the treatment of ALS.

## 7. Conclusion

At present, the pathogenesis of ALS is not fully understood, and the genetic factors are suggested to be closely related to the pathogenesis of ALS. The imbalance of protein homeostasis, the RNA metabolism disorder, the mitochondrial dysfunction, the glutamate-mediated excitatory toxicity and the intra-neuronal material transport disorder in neurons are currently recognized as the identifiable molecular causes of ALS pathogenesis. With the investigated progress of genetics and molecular mechanisms about the pathogenesis of ALS, a deeper understanding about the molecular genetic pathogenesis of ALS has been gained. The mutations in genes such as *SOD1*, *C9ORF72*, *TDP-43*, *ubiquilin-2*, and *VCP* can lead to the protein homeostasis imbalance via the following molecular and/or biological pathways in the pathogenesis of ALS. These gene mutations can interfere with UPS and autophagy degradation pathways, leading to the abnormal accumulation of toxic proteins and ultimately cell death. Both FUS and TDP-43 are RBPs associated with the RNA metabolism pathways, and their mutations can cause the FUS and TDP-43 abnormal localization and aggregation in cells, affecting both RNA metabolism and cell functions and ultimately leading to neurodegeneration in the pathogenesis of ALS. The *FUS* mutations mainly cause the mislocalization of FUS in cytoplasm, resulting in the FUS immunoreactive inclusion bodies. The abnormal RNA splicing pathway caused by the *TDP-43* mutation or the nuclear *TDP-43* depletion promotes the pathways of ALS occurrence through the TDP-43 functional loss and/or toxicity enhancement. The mitochondrial dysfunction plays a crucial role in the pathogenesis of ALS because it affects multiple cellular pathways, including the energy production and the Ca^2+^ regulation, apoptosis, and the protein quality control. The ALS pathogenic genes, such as *TDP-43*, *FUS*, *SOD1*, *C9ORF72*, and *Sigma-1R* mutations, can alter the mitochondrial integrity and function. Both TDP-43 and FUS mutant isoforms, respectively, disrupt the mitochondrial RNA expression and interact with the mitochondrial ATP synthase catalytic subunits, resulting in the abnormal mitochondrial membrane potential, reducing the ATP production and the oxygen consumption. The SOD1 mutations impair the ability of mitochondria to eliminate ROS. The C9ORF72 mutations increase the concentration of neuronal Ca^2+^ influx and the overexpression of polyGR that causes the mitochondrial dysfunction by altering protein binding patterns on mitochondria. Finally, the mutant Sigma-1R cannot bind to IP3R, leading to the Ca^2+^ imbalance in MAM and exacerbating the related pathways of ER stress-induced neuronal death. The excitotoxicity occurs due to the excessive activation of glutamate receptors, failures to clear the glutamate neurotransmitters, or increases the postsynaptic sensitivity to glutamate. Glutamate transporters in astrocytes, such as EAAT1/GLAST and EAAT2/GLT-1, maintain the optimal extracellular glutamate levels and prevent excitotoxicity. The abnormal post-translational modification pathways of EAAT1/GLAST and EAAT2/GLT-1 are associated with the pathogenesis of ALS via the following pathways. The *SOD1* mutant mice enhance the caspase-3-mediated cleavage of EAAT2 (GLT-1), resulting in impairing the glutamate uptake and producing excitotoxicity in ALS patients. On the other hand, *C9ORF72* mutations lead to the production of toxic dipeptide repeat sequences, increasing the expression and Ca^2+^ permeability of AMPA-type glutamate receptors (GluA1), leading to excitotoxicity in motor neurons. Motor neurons depend on the axonal transport pathways to maintain their normal structure and function, and *C9ORF72*, *TDP-43*, and *FUS* mutations are associated with the neuronal transport disorders. The *C9ORF72* gene mutations can affect the vesicular transport pathway, reduce the protein degradation, and cause the neurodegeneration and death of neurons in the pathogenesis of ALS. The *TDP-43* gene deletion reduces the signaling pathways for the epidermal growth factor receptors and the serine/threonine kinase receptors, affecting the neuron survival and axon innervation. In addition, it also interferences with the material circulation and target protein transport pathways on the neuronal cell membrane, ultimately damaging neurons in the pathogenesis of ALS.

In addition, the same genetic mutation may have effects on multiple disease pathways. Currently, conformational and functional variations in SOD1 caused by mutations have been suggested to confer toxicity via the interactions of several proteins and multiple mechanisms that are mutually compatible pathogenic mechanisms. These mechanisms include protein aggregation, mitochondrial dysfunction, oxidative stress, apoptosis, excitotoxicity, and ER stress, etc. (Hayashi et al., 2016; Kaur et al., 2016). *TARDBP* mutations are inherited in an autosomal dominant pattern. Among the various heterodimeric proteins encoded by *TARDBP*, TDP-43 remains the most common (Alsultan et al., 2016). TDP-43 exhibits both nuclear localization and export signals, allowing it to shuttle between the cytoplasm and the nucleus. As a regulator of gene expression, TDP-43 is involved in the regulation of thousands of genes through RNA splicing, DNA or RNA binding, and protein–protein interactions. It also plays a crucial role in several steps of RNA processing, including mRNA stability regulation, mRNA trafficking, pre-mRNA splicing, and regulation of non-coding RNA translation or function (Heyburn and Moussa, 2017). Currently, ALS-associated *FUS* mutations have been shown to cause toxicity by acquiring functional effects in the cytoplasm. Mutant FUS accumulates along the axons, leading to localized stress responses that inhibit local protein synthesis. This includes the suppression of RNA-encoded proteins critical for synaptic function and the inhibition of translation of axonally localized proteins. This process ultimately impairs neuronal synaptic function (López-Erauskin et al., 2020). Overexpression of mutant *FUS* variants, leads to mitochondrial dysfunction through a toxic gain-of-function mechanism, resulting in reduced mitochondrial membrane potential and respiratory function (Tsai et al., 2020). Additionally, the loss or mutation of *C9ORF72* is associated with a range of functional impairments, including synaptic dysfunction, mitochondrial dysfunction, nucleocytoplasmic transport dysfunction, and varying levels of autophagy-lysosomal dysfunction (Beckers et al., 2021; Pang and Hu, 2021; Jiang et al., 2022). Overall, *C9ORF72* plays a critical role in regulating the autophagy-lysosomal pathway, neuroinflammation, SGs formation, lipid metabolism, axonal growth and transport, as well as presynaptic and postsynaptic functions (Pang and Hu, 2021). It is believed that in the near future, the accurate pathogenesis of ALS disease will be clarified, thus alleviating the suffering of patients, extending their lives, improving their quality of life, and even cure this disease.

## Author contributions

WZ wrote the first draft of the manuscript. RX provided critical feedback as supervisor. All authors contributed to the article and approved the submitted version.

## Funding

This study was in part funded by the National Natural Science Foundation of China (30560042, 81160161, 81360198, and 82160255), Education Department of Jiangxi Province (GJJ13198 and GJJ170021), Jiangxi Provincial Department of Science and Technology (20192BAB205043) and Health and Family Planning Commission of Jiangxi Province (20181019 and 202210002).

## Conflict of interest

The authors declare that the research was conducted in the absence of any commercial or financial relationships that could be construed as a potential conflict of interest.

## Publisher’s note

All claims expressed in this article are solely those of the authors and do not necessarily represent those of their affiliated organizations, or those of the publisher, the editors and the reviewers. Any product that may be evaluated in this article, or claim that may be made by its manufacturer, is not guaranteed or endorsed by the publisher.

## Figures and Tables

**Table 1:** 

ALS	amyotrophic lateral sclerosis
AMPAR	alpha-amino-3-hydroxy-5-methyl-4-isoxazolepropionic acid-type ionotropic glutamate receptors
ATP	adenosine triphosphate
CNS	central nervous system
EAAT1	excitatory amino acid transporters 1
EAAT2	excitatory amino acid transporters 2
GLAST	glutamate aspartate transporter
GLT-1	glial glutamate transporter-1
M6PRs	mannose-6-phosphate receptors
MAM	mitochondria-associated endoplasmic reticulum membranes
polyGR	polyglycine-arginine
RBPs	RNA-binding proteins
ROS	reactive oxygen species
UPS	ubiquitin-proteasome system
VAPB	vesicle-associated membrane protein-associated protein B
VDAC1	voltage-dependent anion channel-1

## References

[ref1] Alsultan A. A. Waller R. Heath P. R. Kirby J. (2016). The genetics of amyotrophic lateral sclerosis: current insights. Degener. Neurol. Neuromuscul. Dis. 6, 49–64. doi: 10.2147/DNND.S84956 30050368 PMC6053097

[ref2] Aluri K. C. Salisbury J. P. Prehn J. H. M. Agar J. N. (2020). Loss of angiogenin function is related to earlier ALS onset and a paradoxical increase in ALS duration. Sci. Rep. 10:3715. doi: 10.1038/s41598-020-60431-6, PMID: 32111867 PMC7048737

[ref3] Baradaran-Heravi Y. Van Broeckhoven C. van der Zee J. (2020). Stress granule mediated protein aggregation and underlying gene defects in the FTD-ALS spectrum. Neurobiol. Dis. 134:104639. doi: 10.1016/j.nbd.2019.104639, PMID: 31626953

[ref4] Baron D. M. Fenton A. R. Saez-Atienzar S. Giampetruzzi A.. (2022). ALS-associated KIF5A mutations abolish autoinhibition resulting in a toxic gain of function. Cell Rep. 39:110598. doi: 10.1016/j.celrep.2022.110598, PMID: 35385738 PMC9134378

[ref5] Becker L. A. Huang B. Bieri G. Ma R. Knowles D. A. Jafar-Nejad P.. (2017). Therapeutic reduction of ataxin-2 extends lifespan and reduces pathology in TDP-43 mice. Nature 544, 367–371. doi: 10.1038/nature22038, PMID: 28405022 PMC5642042

[ref6] Beckers J. Tharkeshwar A. K. Van Damme P. (2021). C9orf72 ALS-FTD: recent evidence for dysregulation of the autophagy-lysosome pathway at multiple levels. Autophagy 17, 3306–3322. doi: 10.1080/15548627.2021.1872189, PMID: 33632058 PMC8632097

[ref7] Beijer D. Kim H. J. Guo L. O’Donovan K. Mademan I.. (2021). Characterization of HNRNPA1 mutations defines diversity in pathogenic mechanisms and clinical presentation. JCI Insight 6:148363. doi: 10.1172/jci.insight.148363, PMID: 34291734 PMC8410042

[ref8] Benson B. C. Shaw P. J. Azzouz M. Highley J. R. Hautbergue G. M. (2021). Proteinopathies as hallmarks of impaired gene expression, proteostasis and mitochondrial function in amyotrophic lateral sclerosis. Front. Neurosci. 15:783624. doi: 10.3389/fnins.2021.783624, PMID: 35002606 PMC8733206

[ref9] Boyman L. Karbowski M. Lederer W. J. (2020). Regulation of mitochondrial ATP production: Ca^2+^ signaling and quality control. Trends Mol. Med. 26, 21–39. doi: 10.1016/j.molmed.2019.10.007, PMID: 31767352 PMC7921598

[ref10] Burk K. Pasterkamp R. J. (2019). Disrupted neuronal trafficking in amyotrophic lateral sclerosis. Acta Neuropathol. 137, 859–877. doi: 10.1007/s00401-019-01964-7, PMID: 30721407 PMC6531423

[ref11] Burstein S. R. Valsecchi F. Kawamata H. Bourens M. Zeng R. Zuberi A.. (2018). In vitro and in vivo studies of the ALS-FTLD protein CHCHD10 reveal novel mitochondrial topology and protein interactions. Hum. Mol. Genet. 27, 160–177. doi: 10.1093/hmg/ddx397, PMID: 29112723 PMC5886281

[ref12] Cali C. P. Patino M. Tai Y. K. Ho W. Y. McLean C. A. Morris C. M.. (2019). C9orf72 intermediate repeats are associated with corticobasal degeneration, increased C9orf72 expression and disruption of autophagy. Acta Neuropathol. 138, 795–811. doi: 10.1007/s00401-019-02045-5, PMID: 31327044 PMC6802287

[ref13] Candelise N. Salvatori I. Scaricamazza S. Nesci V. Zenuni H. Ferri A.. (2022). Mechanistic insights of mitochondrial dysfunction in amyotrophic lateral sclerosis: an update on a lasting relationship. Meta 12:233. doi: 10.3390/metabo12030233, PMID: 35323676 PMC8951432

[ref14] Cao B. Gu X. Wei Q. Li C. Chen Y. Ou R.. (2021). Mutation screening and burden analysis of GLT8D1 in Chinese patients with amyotrophic lateral sclerosis. Neurobiol. Aging 101, 298.e17–298.e21. doi: 10.1016/j.neurobiolaging.2020.10.017, PMID: 33581933

[ref15] Chou C.-C. Zhang Y. Umoh M. E. Vaughan S. W. Lorenzini I. Liu F.. (2018). TDP-43 pathology disrupts nuclear pore complexes and nucleocytoplasmic transport in ALS/FTD. Nat. Neurosci. 21, 228–239. doi: 10.1038/s41593-017-0047-3 29311743 PMC5800968

[ref16] Chua J. P. De Calbiac H. Kabashi E. Barmada S. J. (2022). Autophagy and ALS: mechanistic insights and therapeutic implications. Autophagy 18, 254–282. doi: 10.1080/15548627.2021.1926656, PMID: 34057020 PMC8942428

[ref17] Cooper-Knock J. Moll T. Ramesh T. Castelli L. Beer A. Robins H.. (2019). Mutations in the glycosyltransferase domain of GLT8D1 are associated with familial amyotrophic lateral sclerosis. Cell Rep. 26, 2298–2306.e5. doi: 10.1016/j.celrep.2019.02.006, PMID: 30811981 PMC7003067

[ref18] Cull-Candy S. G. Farrant M. (2021). Ca^2+^-permeable AMPA receptors and their auxiliary subunits in synaptic plasticity and disease. J. Physiol. 599, 2655–2671. doi: 10.1113/JP279029, PMID: 33533533 PMC8436767

[ref19] Dafinca R. Barbagallo P. Talbot K. (2021). The role of mitochondrial dysfunction and ER stress in TDP-43 and C9ORF72 ALS. Front. Cell. Neurosci. 15:653688. doi: 10.3389/fncel.2021.653688, PMID: 33867942 PMC8047135

[ref20] Delic V. Kurien C. Cruz J. Zivkovic S. Barretta J. Thomson A.. (2018). Discrete mitochondrial aberrations in the spinal cord of sporadic ALS patients. J. Neurosci. Res. 96, 1353–1366. doi: 10.1002/jnr.24249, PMID: 29732581

[ref21] Deng J. Wang P. Chen X. Cheng H. Liu J. Fushimi K.. (2018). FUS interacts with ATP synthase beta subunit and induces mitochondrial unfolded protein response in cellular and animal models. Proc. Natl. Acad. Sci. U. S. A. 115, E9678–E9686. doi: 10.1073/pnas.1806655115, PMID: 30249657 PMC6187197

[ref22] Deshaies J.-E. Shkreta L. Moszczynski A. J. Sidibé H. Semmler S. Fouillen A.. (2018). TDP-43 regulates the alternative splicing of hnRNP A1 to yield an aggregation-prone variant in amyotrophic lateral sclerosis. Brain 141, 1320–1333. doi: 10.1093/brain/awy062, PMID: 29562314 PMC5917749

[ref23] Diaper D. C. Adachi Y. Lazarou L. Greenstein M. Simoes F. A. Di Domenico A.. (2013). Drosophila TDP-43 dysfunction in glia and muscle cells cause cytological and behavioural phenotypes that characterize ALS and FTLD. Hum. Mol. Genet. 22, 3883–3893. doi: 10.1093/hmg/ddt243 23727833 PMC3766182

[ref24] Farrawell N. E. Yerbury J. J. (2021). Mutant cu/Zn superoxide dismutase (A4V) turnover is altered in cells containing inclusions. Front. Mol. Neurosci. 14:771911. doi: 10.3389/fnmol.2021.771911, PMID: 34803609 PMC8597841

[ref25] Fernández-Ruiz J. de Lago E. Rodríguez-Cueto C. Moro M. A. (2021). Recent advances in the pathogenesis and therapeutics of amyotrophic lateral sclerosis. Br. J. Pharmacol. 178, 1253–1256. doi: 10.1111/bph.15348 33638898

[ref26] Fiscon G. Conte F. Amadio S. Volonté C. Paci P. (2021). Drug repurposing: a network-based approach to amyotrophic lateral sclerosis. Neurotherapeutics 18, 1678–1691. doi: 10.1007/s13311-021-01064-z 33987813 PMC8609089

[ref27] Gautam M. Xie E. F. Kocak N. Ozdinler P. H. (2019). Mitoautophagy: a unique self-destructive path mitochondria of upper motor neurons with TDP-43 pathology take, very early in ALS. Front. Cell. Neurosci. 13:489. doi: 10.3389/fncel.2019.00489, PMID: 31787882 PMC6854036

[ref28] Gendron T. F. Petrucelli L. (2018). Disease mechanisms of C9ORF72 repeat expansions. Cold Spring Harb. Perspect. Med. 8:a024224. doi: 10.1101/cshperspect.a024224, PMID: 28130314 PMC5880161

[ref29] Hallegger M. Chakrabarti A. M. Lee F. C. Y. Lee B. L. Amalietti A. G. Odeh H. M.. (2021). TDP-43 condensation properties specify its RNA-binding and regulatory repertoire. Cells 184, 4680–4696.e22. doi: 10.1016/j.cell.2021.07.018, PMID: 34380047 PMC8445024

[ref30] Hayashi Y. Homma K. Ichijo H. (2016). SOD1 in neurotoxicity and its controversial roles in SOD1 mutation-negative ALS. Adv Biol Regul 60, 95–104. doi: 10.1016/j.jbior.2015.10.006, PMID: 26563614

[ref31] He J. Liu X. Tang L. Zhao C. He J. Fan D. (2020). Whole-exome sequencing identified novel KIF5A mutations in Chinese patients with amyotrophic lateral sclerosis and Charcot-Marie-tooth type 2. J. Neurol. Neurosurg. Psychiatry 91, 326–328. doi: 10.1136/jnnp-2019-320483 31422367

[ref32] Herrando-Grabulosa M. Gaja-Capdevila N. Vela J. M. Navarro X. (2021). Sigma 1 receptor as a therapeutic target for amyotrophic lateral sclerosis. Br. J. Pharmacol. 178, 1336–1352. doi: 10.1111/bph.15224, PMID: 32761823

[ref33] Heyburn L. Moussa C. E.-H. (2017). TDP-43 in the spectrum of MND-FTLD pathologies. Mol. Cell. Neurosci. 83, 46–54. doi: 10.1016/j.mcn.2017.07.001, PMID: 28687523 PMC5581706

[ref34] Hirschberg S. Dvorzhak A. Rasooli-Nejad S. M. A. Angelov S. Kirchner M. Mertins P.. (2021). Uncoupling the excitatory amino acid transporter 2 from its C-terminal interactome restores synaptic glutamate clearance at corticostriatal synapses and alleviates mutant huntingtin-induced hypokinesia. Front. Cell. Neurosci. 15:792652. doi: 10.3389/fncel.2021.792652 35173582 PMC8841566

[ref35] Humphrey J. Birsa N. Milioto C. McLaughlin M. Ule A. M. Robaldo D.. (2020). FUS ALS-causative mutations impair FUS autoregulation and splicing factor networks through intron retention. Nucl. Acids Res. 48, 6889–6905. doi: 10.1093/nar/gkaa410, PMID: 32479602 PMC7337901

[ref36] Je G. Keyhanian K. Ghasemi M. (2021). Overview of stem cells therapy in amyotrophic lateral sclerosis. Neurol. Res. 43, 616–632. doi: 10.1080/01616412.2021.1893564 33632084

[ref37] Jiang L. Zhang T. Lu K. Qi S. (2022). The progress in C9orf72 research: ALS/FTD pathogenesis, functions and structure. Small GTPases 13, 56–76. doi: 10.1080/21541248.2021.1892443 33663328 PMC9707547

[ref38] Johnson J. O. Chia R. Miller D. E. Li R. Kumaran R. Abramzon Y.. (2021). Association of Variants in the SPTLC1 gene with juvenile amyotrophic lateral sclerosis. JAMA Neurol. 78, 1236–1248. doi: 10.1001/jamaneurol.2021.2598, PMID: 34459874 PMC8406220

[ref39] Johnson J. Pajarillo E. Karki P. Kim J. Son D.-S. Aschner M.. (2018). Valproic acid attenuates manganese-induced reduction in expression of GLT-1 and GLAST with concomitant changes in murine dopaminergic neurotoxicity. Neurotoxicology 67, 112–120. doi: 10.1016/j.neuro.2018.05.001, PMID: 29778792 PMC6441963

[ref40] Kaur S. J. McKeown S. R. Rashid S. (2016). Mutant SOD1 mediated pathogenesis of amyotrophic lateral sclerosis. Gene 577, 109–118. doi: 10.1016/j.gene.2015.11.049 26657039

[ref41] Khani M. Alavi A. Shamshiri H. Zamani B. Hassanpour H. Kazemi M. H.. (2019). Mutation screening of SLC52A3, C19orf12, and TARDBP in Iranian ALS patients. Neurobiol. Aging 75, 225.e9–225.e14. doi: 10.1016/j.neurobiolaging.2018.11.003, PMID: 30553531

[ref42] Kim G. Gautier O. Tassoni-Tsuchida E. Ma X. R. Gitler A. D. (2020). ALS genetics: gains, losses, and implications for future therapies. Neuron 108, 822–842. doi: 10.1016/j.neuron.2020.08.022, PMID: 32931756 PMC7736125

[ref43] Kim J. Liao Y.-H. Ionita C. Bale A. E. Darras B. Acsadi G. (2016). Mitochondrial membrane protein-associated neurodegeneration mimicking juvenile amyotrophic lateral sclerosis. Pediatr. Neurol. 64, 83–86. doi: 10.1016/j.pediatrneurol.2016.08.013, PMID: 27671242

[ref44] Kim H. J. Taylor J. P. (2017). Lost in transportation: nucleocytoplasmic transport defects in ALS and other neurodegenerative diseases. Neuron 96, 285–297. doi: 10.1016/j.neuron.2017.07.029, PMID: 29024655 PMC5678982

[ref45] Klim J. R. Pintacuda G. Nash L. A. Guerra San Juan I. Eggan K. (2021). Connecting TDP-43 pathology with neuropathy. Trends Neurosci. 44, 424–440. doi: 10.1016/j.tins.2021.02.008, PMID: 33832769

[ref46] Korobeynikov V. A. Lyashchenko A. K. Blanco-Redondo B. Jafar-Nejad P. Shneider N. A. (2022). Antisense oligonucleotide silencing of FUS expression as a therapeutic approach in amyotrophic lateral sclerosis. Nat. Med. 28, 104–116. doi: 10.1038/s41591-021-01615-z, PMID: 35075293 PMC8799464

[ref47] Kundra R. Dobson C. M. Vendruscolo M. (2020). A cell-and tissue-specific weakness of the protein homeostasis system underlies brain vulnerability to protein aggregation. iScience 23:100934. doi: 10.1016/j.isci.2020.100934, PMID: 32146327 PMC7063235

[ref48] Lapucci A. Cavone L. Buonvicino D. Felici R. Gerace E. Zwergel C.. (2017). Effect of class II HDAC inhibition on glutamate transporter expression and survival in SOD1-ALS mice. Neurosci. Lett. 656, 120–125. doi: 10.1016/j.neulet.2017.07.033, PMID: 28732762

[ref49] Lee S. Jeon Y.-M. Cha S.-J. Kim S. Kwon Y. Jo M.. (2020). PTK2/FAK regulates UPS impairment via SQSTM1/p62 phosphorylation in TARDBP/TDP-43 proteinopathies. Autophagy 16, 1396–1412. doi: 10.1080/15548627.2019.1686729 31690171 PMC7469499

[ref50] Lee S. Kwon Y. Kim S. Jo M. Jeon Y.-M. Cheon M.. (2020). The role of HDAC6 in TDP-43-induced neurotoxicity and UPS impairment. Front. Cell Dev. Biol. 8:581942. doi: 10.3389/fcell.2020.581942, PMID: 33282865 PMC7705063

[ref51] Leskelä S. Huber N. Rostalski H. Natunen T. Remes A. M. Takalo M.. (2019). C9orf72 proteins regulate autophagy and undergo autophagosomal or proteasomal degradation in a cell type-dependent manner. Cells 8:1233. doi: 10.3390/cells8101233, PMID: 31658762 PMC6829620

[ref52] Li C. Hou Y. Wei Q. Lin J. Jiang Z. Jiang Q.. (2023). Mutation screening of SPTLC1 and SPTLC2 in amyotrophic lateral sclerosis. Hum. Genomics 17:28. doi: 10.1186/s40246-023-00479-3 36966328 PMC10040122

[ref53] Ling S.-C. (2018). Synaptic paths to neurodegeneration: the emerging role of TDP-43 and FUS in synaptic functions. Neural Plast. 2018, 1–13. doi: 10.1155/2018/8413496 PMC5925147 29755516

[ref54] Ling S.-C. Polymenidou M. Cleveland D. W. (2013). Converging mechanisms in ALS and FTD: disrupted RNA and protein homeostasis. Neuron 79, 416–438. doi: 10.1016/j.neuron.2013.07.033, PMID: 23931993 PMC4411085

[ref55] Liu X. Pimm M. L. Haarer B. Brawner A. T. Henty-Ridilla J. L. (2022). Biochemical characterization of actin assembly mechanisms with ALS-associated profilin variants. Eur. J. Cell Biol. 101:151212. doi: 10.1016/j.ejcb.2022.151212, PMID: 35248815 PMC10163920

[ref56] López-Erauskin J. Tadokoro T. Baughn M. W. Myers B. McAlonis-Downes M. Chillon-Marinas C.. (2020). ALS/FTD-linked mutation in FUS suppresses intra-axonal protein synthesis and drives disease without nuclear loss-of-function of FUS. Neuron 106:354. doi: 10.1016/j.neuron.2020.04.006, PMID: 32325059 PMC7184958

[ref57] Machts J. Vielhaber S. Kollewe K. Petri S. Kaufmann J. Schoenfeld M. A. (2018). Global hippocampal volume reductions and local CA1 shape deformations in amyotrophic lateral sclerosis. Front. Neurol. 9:565. doi: 10.3389/fneur.2018.00565, PMID: 30079050 PMC6062964

[ref58] Mackenzie I. R. Nicholson A. M. Sarkar M. Messing J. Purice M. D. Pottier C.. (2017). TIA1 mutations in amyotrophic lateral sclerosis and frontotemporal dementia promote phase separation and Alter stress granule dynamics. Neuron 95, 808–816.e9. doi: 10.1016/j.neuron.2017.07.025, PMID: 28817800 PMC5576574

[ref59] Maurel C. Dangoumau A. Marouillat S. Brulard C. Chami A. Hergesheimer R.. (2018). Causative genes in amyotrophic lateral sclerosis and protein degradation pathways: a link to neurodegeneration. Mol. Neurobiol. 55, 6480–6499. doi: 10.1007/s12035-017-0856-0 29322304

[ref60] Mohassel P. Donkervoort S. Lone M. A. Nalls M. Gable K. Gupta S. D.. (2021). Childhood amyotrophic lateral sclerosis caused by excess sphingolipid synthesis. Nat. Med. 27, 1197–1204. doi: 10.1038/s41591-021-01346-1, PMID: 34059824 PMC9309980

[ref61] Muñoz-Lasso D. C. Romá-Mateo C. Pallardó F. V. Gonzalez-Cabo P. (2020). Much more than a scaffold: cytoskeletal proteins in neurological disorders. Cells 9:358. doi: 10.3390/cells9020358, PMID: 32033020 PMC7072452

[ref62] Nassif M. Woehlbier U. Manque P. A. (2017). The enigmatic role of C9ORF72 in autophagy. Front. Neurosci. 11:442. doi: 10.3389/fnins.2017.00442, PMID: 28824365 PMC5541066

[ref63] Neelagandan N. Gonnella G. Dang S. Janiesch P. C. Miller K. K. Küchler K.. (2019). TDP-43 enhances translation of specific mRNAs linked to neurodegenerative disease. Nucl. Acids Res. 47, 341–361. doi: 10.1093/nar/gky972, PMID: 30357366 PMC6326785

[ref64] Olloquequi J. Cornejo-Córdova E. Verdaguer E. Soriano F. X. Binvignat O. Auladell C.. (2018). Excitotoxicity in the pathogenesis of neurological and psychiatric disorders: therapeutic implications. J. Psychopharmacol. 32, 265–275. doi: 10.1177/0269881118754680 29444621

[ref65] Osking Z. Ayers J. I. Hildebrandt R. Skruber K. Brown H. Ryu D.. (2019). ALS-linked SOD1 mutants enhance neurite outgrowth and branching in adult motor neurons. iScience 11, 294–304. doi: 10.1016/j.isci.2018.12.026, PMID: 30639851 PMC6327879

[ref66] Pajarillo E. Rizor A. Lee J. Aschner M. Lee E. (2019). The role of astrocytic glutamate transporters GLT-1 and GLAST in neurological disorders: potential targets for neurotherapeutics. Neuropharmacology 161:107559. doi: 10.1016/j.neuropharm.2019.03.002, PMID: 30851309 PMC6731169

[ref67] Pang W. Hu F. (2021). Cellular and physiological functions of C9ORF72 and implications for ALS/FTD. J. Neurochem. 157, 334–350. doi: 10.1111/jnc.15255, PMID: 33259633 PMC8842544

[ref68] Pimm M. L. Hotaling J. Henty-Ridilla J. L. (2020). Profilin choreographs actin and microtubules in cells and cancer. Int. Rev. Cell Mol. Biol. 355:5. doi: 10.1016/bs.ircmb.2020.05.005, PMID: 32859370 PMC7461721

[ref69] Pinto-Costa R. Sousa S. C. Leite S. C. Nogueira-Rodrigues J. Ferreira da Silva T. Machado D.. (2020). Profilin 1 delivery tunes cytoskeletal dynamics toward CNS axon regeneration. J. Clin. Invest. 130, 2024–2040. doi: 10.1172/JCI125771, PMID: 31945017 PMC7108904

[ref70] Prehn J. H. M. Jirström E. (2020). Angiogenin and tRNA fragments in Parkinson’s disease and neurodegeneration. Acta Pharmacol. Sin. 41, 442–446. doi: 10.1038/s41401-020-0375-9, PMID: 32144338 PMC7470775

[ref71] Purandare N. Somayajulu M. Hüttemann M. Grossman L. I. Aras S. (2018). The cellular stress proteins CHCHD10 and MNRR1 (CHCHD2): partners in mitochondrial and nuclear function and dysfunction. J. Biol. Chem. 293, 6517–6529. doi: 10.1074/jbc.RA117.001073, PMID: 29540477 PMC5925800

[ref72] Rosenblum L. T. Shamamandri-Markandaiah S. Ghosh B. Foran E. Lepore A. C. Pasinelli P.. (2017). Mutation of the caspase-3 cleavage site in the astroglial glutamate transporter EAAT2 delays disease progression and extends lifespan in the SOD1-G93A mouse model of ALS. Exp. Neurol. 292, 145–153. doi: 10.1016/j.expneurol.2017.03.014, PMID: 28342750 PMC5433801

[ref73] Sahadevan S. Hembach K. M. Tantardini E. Pérez-Berlanga M. Hruska-Plochan M. Megat S.. (2021). Synaptic FUS accumulation triggers early misregulation of synaptic RNAs in a mouse model of ALS. Nat. Commun. 12:3027. doi: 10.1038/s41467-021-23188-8 34021139 PMC8140117

[ref74] Sakai S. Watanabe S. Komine O. Sobue A. Yamanaka K. (2021). Novel reporters of mitochondria-associated membranes (MAM), MAMtrackers, demonstrate MAM disruption as a common pathological feature in amyotrophic lateral sclerosis. FASEB J. 35:e21688. doi: 10.1096/fj.202100137R, PMID: 34143516

[ref75] Schmidt E. J. Funes S. McKeon J. E. Morgan B. R. Boopathy S. O’Connor L. C.. (2021). ALS-linked PFN1 variants exhibit loss and gain of functions in the context of formin-induced actin polymerization. Proc. Natl. Acad. Sci. U. S. A. 118:e2024605118. doi: 10.1073/pnas.2024605118, PMID: 34074767 PMC8201830

[ref76] Sekiyama N. Takaba K. Maki-Yonekura S. Akagi K.-I. Ohtani Y. Imamura K.. (2022). ALS mutations in the TIA-1 prion-like domain trigger highly condensed pathogenic structures. Proc. Natl. Acad. Sci. U. S. A. 119:e2122523119. doi: 10.1073/pnas.2122523119, PMID: 36112647 PMC9499527

[ref77] Semmler S. Gagné M. Garg P. Pickles S. R. Baudouin C. Hamon-Keromen E.. (2020). TNF receptor-associated factor 6 interacts with ALS-linked misfolded superoxide dismutase 1 and promotes aggregation. J. Biol. Chem. 295, 3808–3825. doi: 10.1074/jbc.RA119.011215, PMID: 32029478 PMC7086032

[ref78] Shenouda M. Zhang A. B. Weichert A. Robertson J. (2018). Mechanisms associated with TDP-43 neurotoxicity in ALS/FTLD. Adv Neurobiol 20, 239–263. doi: 10.1007/978-3-319-89689-2_9, PMID: 29916022

[ref79] Shi Y. Hung S.-T. Rocha G. Lin S. Linares G. R. Staats K. A.. (2019). Identification and therapeutic rescue of autophagosome and glutamate receptor defects in C9ORF72 and sporadic ALS neurons. JCI Insight 4:127736. doi: 10.1172/jci.insight.127736 PMC6693831 31310593

[ref80] Shi Y. Lin S. Staats K. A. Li Y. Chang W.-H. Hung S.-T.. (2018). Haploinsufficiency leads to neurodegeneration in C9ORF72 ALS/FTD human induced motor neurons. Nat. Med. 24, 313–325. doi: 10.1038/nm.4490, PMID: 29400714 PMC6112156

[ref81] Shoshan-Barmatz V. Shteinfer-Kuzmine A. Verma A. (2020). VDAC1 at the intersection of cell metabolism, apoptosis, and diseases. Biomol. Ther. 10:1485. doi: 10.3390/biom10111485, PMID: 33114780 PMC7693975

[ref82] Smith E. F. Shaw P. J. De Vos K. J. (2019). The role of mitochondria in amyotrophic lateral sclerosis. Neurosci. Lett. 710:132933. doi: 10.1016/j.neulet.2017.06.052 28669745

[ref83] Starr A. Sattler R. (2018). Synaptic dysfunction and altered excitability in C9ORF72 ALS/FTD. Brain Res. 1693, 98–108. doi: 10.1016/j.brainres.2018.02.011 29453960 PMC5997509

[ref84] Straub I. R. Janer A. Weraarpachai W. Zinman L. Robertson J. Rogaeva E.. (2018). Loss of CHCHD10-CHCHD2 complexes required for respiration underlies the pathogenicity of a CHCHD10 mutation in ALS. Hum. Mol. Genet. 27, 178–189. doi: 10.1093/hmg/ddx393, PMID: 29121267 PMC5886208

[ref85] Tedesco B. Ferrari V. Cozzi M. Chierichetti M. Casarotto E. Pramaggiore P.. (2022). The role of small heat shock proteins in protein misfolding associated motoneuron diseases. Int. J. Mol. Sci. 23:11759. doi: 10.3390/ijms231911759, PMID: 36233058 PMC9569637

[ref86] Tedesco B. Vendredy L. Timmerman V. Poletti A. (2023). The chaperone-assisted selective autophagy complex dynamics and dysfunctions. Autophagy 19, 1619–1641. doi: 10.1080/15548627.2022.2160564, PMID: 36594740 PMC10262806

[ref87] Tsai Y.-L. Coady T. H. Lu L. Zheng D. Alland I. Tian B.. (2020). ALS/FTD-associated protein FUS induces mitochondrial dysfunction by preferentially sequestering respiratory chain complex mRNAs. Genes Dev. 34, 785–805. doi: 10.1101/gad.335836.119, PMID: 32381627 PMC7263147

[ref88] Vant Spijker H. M. Almeida S. (2023). How villains are made: the translation of dipeptide repeat proteins in C9ORF72-ALS/FTD. Gene 858:147167. doi: 10.1016/j.gene.2023.147167, PMID: 36621656 PMC9928902

[ref89] Wang W. Arakawa H. Wang L. Okolo O. Siedlak S. L. Jiang Y.. (2017). Motor-coordinative and cognitive dysfunction caused by mutant TDP-43 could be reversed by inhibiting its mitochondrial localization. Mol. Ther. 25, 127–139. doi: 10.1016/j.ymthe.2016.10.013, PMID: 28129109 PMC5363201

[ref90] Wang P. Deng J. Dong J. Liu J. Bigio E. H. Mesulam M.. (2019). TDP-43 induces mitochondrial damage and activates the mitochondrial unfolded protein response. PLoS Genet. 15:e1007947. doi: 10.1371/journal.pgen.1007947, PMID: 31100073 PMC6524796

[ref91] Wani A. Weihl C. C. (2021). Loss-of-function mutation in VCP mimics the characteristic pathology as in FTLD-TARDBP. Autophagy 17, 4502–4503. doi: 10.1080/15548627.2021.1985880, PMID: 34632910 PMC8726662

[ref92] Wood A. Gurfinkel Y. Polain N. Lamont W. Rea S. Y. (2021). Molecular mechanisms underlying TDP-43 pathology in cellular and animal models of ALS and FTLD. Int. J. Mol. Sci. 22:4705. doi: 10.3390/ijms22094705 33946763 PMC8125728

[ref93] Wu J. J. Cai A. Greenslade J. E. Higgins N. R. Fan C. Le N. T. T.. (2020). ALS/FTD mutations in UBQLN2 impede autophagy by reducing autophagosome acidification through loss of function. Proc. Natl. Acad. Sci. U. S. A. 117, 15230–15241. doi: 10.1073/pnas.1917371117, PMID: 32513711 PMC7334651

[ref94] Wu N.-H. Ye Y. Wan B.-B. Yu Y.-D. Liu C. Chen Q.-J. (2021). Emerging benefits: pathophysiological functions and target drugs of the Sigma-1 receptor in neurodegenerative diseases. Mol. Neurobiol. 58, 5649–5666. doi: 10.1007/s12035-021-02524-5 34383254

[ref95] Xu W. Xu J. (2018). C9orf72 dipeptide repeats cause selective neurodegeneration and cell-autonomous excitotoxicity in drosophila glutamatergic neurons. J. Neurosci. 38, 7741–7752. doi: 10.1523/JNEUROSCI.0908-18.2018, PMID: 30037833 PMC6705968

[ref96] Xue Y. C. Ng C. S. Xiang P. Liu H. Zhang K. Mohamud Y.. (2020). Dysregulation of RNA-binding proteins in amyotrophic lateral sclerosis. Front. Mol. Neurosci. 13:78. doi: 10.3389/fnmol.2020.00078 32547363 PMC7273501

[ref97] Yamashita T. Kwak S. (2019). Cell death cascade and molecular therapy in ADAR2-deficient motor neurons of ALS. Neurosci. Res. 144, 4–13. doi: 10.1016/j.neures.2018.06.004 29944911

[ref98] Yasuda K. Clatterbuck-Soper S. F. Jackrel M. E. Shorter J. Mili S. (2017). FUS inclusions disrupt RNA localization by sequestering kinesin-1 and inhibiting microtubule detyrosination. J. Cell Biol. 216, 1015–1034. doi: 10.1083/jcb.201608022, PMID: 28298410 PMC5379945

[ref99] Yin P. Bai D. Zhu L. Deng F. Guo X. Li B.. (2021). Cytoplasmic TDP-43 impairs the activity of the ubiquitin-proteasome system. Exp. Neurol. 345:113833. doi: 10.1016/j.expneurol.2021.113833, PMID: 34363810

[ref100] Zhang K. Wang A. Zhong K. Qi S. Wei C. Shu X.. (2021). UBQLN2-HSP70 axis reduces poly-Gly-ala aggregates and alleviates behavioral defects in the C9ORF72 animal model. Neuron 109, 1949–1962.e6. doi: 10.1016/j.neuron.2021.04.023 33991504

[ref101] Zhao J. Fok A. H. K. Fan R. Kwan P.-Y. Chan H.-L. Lo L. H.-Y.. (2020). Specific depletion of the motor protein KIF5B leads to deficits in dendritic transport, synaptic plasticity and memory. elife 9:e53456. doi: 10.7554/eLife.53456, PMID: 31961321 PMC7028368

[ref102] Ziff O. J. Clarke B. E. Taha D. M. Crerar H. Luscombe N. M. Patani R. (2022). Meta-analysis of human and mouse ALS astrocytes reveals multi-omic signatures of inflammatory reactive states. Genome Res. 32, 71–84. doi: 10.1101/gr.275939.121, PMID: 34963663 PMC8744676

